# Open Knee(s): A Free and Open Source Library of Specimen-Specific Models and Related Digital Assets for Finite Element Analysis of the Knee Joint

**DOI:** 10.1007/s10439-022-03074-0

**Published:** 2022-09-14

**Authors:** Snehal Chokhandre, Ariel Schwartz, Ellen Klonowski, Benjamin Landis, Ahmet Erdemir

**Affiliations:** Department of Biomedical Engineering, Lerner Research Institute, Cleveland Clinic, Cleveland, OH USA

**Keywords:** Finite element analysis, Musculoskeletal biomechanics, Tibiofemoral joint, Patellofemoral joint, Passive flexion, Simulation, Computational model

## Abstract

There is a growing interest in the use of virtual representations of the knee for musculoskeletal research and clinical decision making, and to generate digital evidence for design and regulation of implants. Accessibility to previously developed models and related digital assets can dramatically reduce barriers to entry to conduct simulation-based studies of the knee joint and therefore help accelerate scientific discovery and clinical innovations. Development of models for finite element analysis is a demanding process that is both time consuming and resource intensive. It necessitates expertise to transform raw data to reliable virtual representations. Modeling and simulation workflow has many processes such as image segmentation, surface geometry generation, mesh generation and finally, creation of a finite element representation with relevant loading and boundary conditions. The outcome of the workflow is not only the end-point knee model but also many other digital by-products. When all of these data, derivate assets, and tools are freely and openly accessible, researchers can bypass some or all the steps required to build models and focus on using them to address their research goals. With provenance to specimen-specific anatomical and mechanical data and traceability of digital assets throughout the whole lifecycle of the model, reproducibility and credibility of the modeling practice can be established. The objective of this study is to disseminate Open Knee(s), a cohort of eight knee models (and relevant digital assets) for finite element analysis, that are based on comprehensive specimen-specific imaging data. In addition, the models and by-products of modeling workflows are described along with model development strategies and tools. Passive flexion served as a test simulation case, demonstrating an end-user application. Potential roadmaps for reuse of Open Knee(s) are also discussed.

**Supplementary Information:**

The online version contains supplementary material available at 10.1007/s10439-022-03074-0.

## Introduction

Computational modeling and simulation of biological structures is ubiquitous from scientific research to clinical decision making. Finite element (FE) analysis in particular can be a transformative tool to advance the understanding of structure–function interactions in biological systems and can also assist in clinical planning or decision making concerning the biomechanics of joints, organs, and medical devices.^16^ Advances and availability of modeling and simulation have made it possible to create increasingly detailed models which can reliably represent the anatomy and physiology for prediction of biomechanical response under a variety of loading scenarios.^5,51^

FE methods have long been used to study joint mechanics, in particular for explorations of the human knee.^12,29^ This is an expected consequence of knee biomechanics being a vast research area and the knee being a site of high incidence and prevalence of musculoskeletal problems. In the United States, there are more than 10 million visits to clinics every year because of knee injury or pathology.^48^ The knee is exposed to high forces during activities of daily living, exercise, and sports performance. Consequently, tissue damage and injuries are frequent and require surgical intervention (such as., anterior cruciate ligament reconstruction^11,32^). Pathological conditions of cartilage are also highly common. Osteoarthritis, for example, impacts more than 27 million people in the United States, causing diminished quality of life, disability, and eventually joint replacement.^31^ Interventional or conservative management of knee problems requires foundational knowledge of the joint’s mechanical function and its tissue structures’ anatomical and mechanical condition. Subsequently, predicting biomechanical consequences of joint and tissue reconstruction is highly desirable. Simulation using virtual knees offers a prominent solution to match these needs.^12,29^

Knee models have been used to explore joint and tissue function,^3,37^ understand injury mechanisms,^30,40^ study pathological joint mechanics,^4,21^ evaluate surgical performance,^42^ prototype tissue reconstruction techniques,^13,38^ and facilitate implant design.^6,27^ Given the anatomical and mechanical complexity of the knee, development of computational knee models can be challenging. Execution of the modeling and simulation workflow requires a good understanding of the geometry of the bones, cartilage, menisci, ligaments, and tendons as well as how these components interact with each other under various conditions.^19^ Additionally, transformation of medical images such as magnetic resonance images (MRI) to a three-dimensional anatomical and mechanical representation, i.e. for finite element analysis, requires several intermediate stages of processing: segmentation to delineate the boundaries of structures, creating closed volumes and meshes, defining regions where tissues connect or interact (contact, for example), assembly of all tissue components, defining material properties, and applying appropriate boundary conditions etc.^41^ This necessitates a prerequisite of understanding and expertise not only in knee anatomy and mechanics but also in various computational tools. In result, delivery of simulation-ready models is a challenging and time consuming task that may necessitate manual intervention by the modeler. Often times, methods and specifications to go through these pipelines are not clearly defined or available in existing literature.^26^ Further, there is a need for associated mechanical data, ideally specimen-specific, not only to develop individualized models but also to assess their predictive capacity.^8,9^ The burden of collecting comprehensive anatomical and mechanical data and the challenges of developing usable models can limit aspiring knee biomechanics scientists and engineers from pursuing their research or innovation goals.

Publicly accessible data, models, guidelines, and tools can greatly alleviate the challenges associated with the process of model development, customization, calibration, and benchmarking.^18^ There has been a concerted effort in making data available freely for biomechanical studies in the last few years.^18^ Sharing and exchange of models and data can facilitate reuse and enhance assessment of reproducibility.^17,18^ Recently, studies such as those done by Strocchi et al.^49^ have provided an impressive detailed mesh dataset for hearts. However, without the associated data such as images, segmentation or further model information, the burden falls on the user to have the resources and expertise to regenerate models for their research and ensure the reliability of the actual geometry. A recent study by Wittek et al.^52^ has provided a fairly extensive dataset for abdominal aortas with image, segmentation, mesh and boundary condition information. The lack of mechanical data associated with it may render model validation challenging. Specifically for knee biomechanics, studies such as the Osteoarthritis Initiative^14,22^ have been instrumental in providing large amounts of heterogeneous clinical data. Mechanical and imaging data sets and models such as those developed at University of Denver^28,35^ so far provide an extensive example of dissemination for building knee models. Nonetheless, intermediate products of modeling and simulation workflow (segmentations, soft tissue continuum meshes) have not been provided and some simplifications have been adopted in building the model components, such as, ligaments were represented by springs and menisci were not included in the model.

To be most effective in assisting others’ research endeavors, it is imperative that all of the intermediate derivative data of the modeling and simulation workflow are provided along with the source data (anatomy and mechanics) and final model.^18^ This would help to establish provenance and based on the context of modeling or need, any stage of the pipeline can be utilized as a starting point. If segmentations, surface geometries, meshes and models are all provided, necessary or desired modifications can be performed on any or all of these data. This will also allow quality assessment at various stages of modeling and support execution of reproducibility studies. With mechanical data, which may be utilized for context of use relevant validation, credibility of models can be enhanced.^19^ Associated specifications incorporating guidelines for building and reporting study details, further strengthen the trust in the provided models and data.^20^ Additionally, appropriately annotated data facilitates findability of components within the specimen or across specimens.^50^

With Open Knee(s), we aim to provide a detailed database of all digital assets generated during development of a cohort of knee models for finite element analysis. Specifically, provenance to readily disseminated mechanical and imaging data^9^ can be established. All products of the modeling workflow (models and intermediate digital assets) are provided: tissue segmentation labels, raw and smooth surface geometries, surface meshes with multiple densities, volume meshes with prerequisite set definitions, template finite element representations of the tibiofemoral and patellofemoral joints, and customized models for simulation of passive flexion. In addition, specifications for the use of free and open source tools for modeling and Python scripts to automate various components of the pipeline are disseminated. This comprehensive dissemination is aimed for inspection and reuse of our knee modeling and simulation workflow and cohort of models, by anyone for any purpose, in support of creating digital evidence for relevant knee biomechanics problems.

## Material and Methods

### Anatomical and Mechanical Data

Comprehensive imaging and joint mechanics data were collected with the goal of supporting the development of high fidelity knee joint models. The extent of data and experiment protocols are briefly summarized here, whereas the details can be found in the previously published data descriptor,^9^ and the data can be publicly accessed at a permanent location.^36^

#### Donor Information

Models were developed for eight knee specimens from cadaver donors. The specimens are representative of target population of males and females including young (18–35 years) with healthy cartilage, middle aged (40–65 years) with healthy cartilage, elderly (65–80 years) with healthy or pathological cartilage. The donors were targeted to be within the following normative ranges: height (1.5–1.8 m), weight (45–90 kg), and a body mass index (BMI, 18.5–24.9). Further demographic details can be found in Table 1. Further information regarding tissue health was provided in the previously published data descriptor.^9^

**TABLE 1:** Donor specifics for Open Knee(s) models.

Specimen ID	oks001	oks002	oks003	oks004	oks006	oks007	oks008	oks009
Side	Right	Right	Left	Right	Right	Right	Right	Left
Gender	Male	Female	Female	Female	Female	Male	Male	Male
Age (years)	71	67	25	46	71	71	40	34
Race	White	White	White	White	White	White	White	White
Height (m)	1.83	1.55	1.73	1.58	1.52	1.70	1.78	1.80
Weight (kg)	77.1	45.3	68.0	54.4	49.4	65.8	63.5	68.03
BMI	23.1	18.9	22.8	21.9	21.3	22.7	20.09	20.0

The information corresponds, and therefore, establishes provenance to that provided in the data descriptor for specimen-specific imaging and joint mechanics

#### Specimen-Specific Imaging Data

Magnetic resonance images were acquired on a 3 Tesla equipment using three protocols: general purpose, cartilage focused, connective tissue focused. These were based on imaging sequences of Osteoarthritis Initiative^39^ and iterated to serve for modeling and simulation purposes, such as, for increased resolution, contrast, and field of view. General purpose imaging aimed at a field of view large enough to capture tibiofemoral and patellofemoral joints and registration markers attached to the bones. The imaging protocol was a 3D T1-weighted sequence without fat suppression (TR = 20, TE = 6; 0.5 mm × 0.5 mm × 0.5 mm). Cartilage imaging relied on a 3D T1-weighted sequence with fat suppression (TR = 29, TE = 5.3; 0.35 mm × 0.35 mm × 0.7 mm). Connective tissue imaging relied on three orthogonal acquisitions in axial, sagittal, and coronal planes. A proton density imaging sequence was used (TR = 10,000, TE = 9.7; 0.35 mm × 0.35 mm × 2.8 mm). This image modality aimed to assist the localization of ligament insertions and boundaries, and meniscus volume. During imaging, the specimens were not moved to ensure the alignment of image volumes. Imaging data were made publicly available as part of a previous effort on data description and dissemination.^9^

#### Specimen-Specific Mechanical Data

The availability of specimen-specific joint mechanics data for the cohort of knees is worth mentioning. While these data were not utilized for model development, they are imperative for prospective validation studies for relevant model contexts of use. Mechanical testing was performed on a robotics testing system. The tibiofemoral joint was characterized by passive flexion, laxity, and combined loading tests. Passive flexion was conducted from 0° to 90° to quantify the characteristic motion of the knee guided by articular contact and ligaments. Laxity tests were conducted at 0°, 30°, 60°, and 90° of flexion and included internal–external rotation (0 to ± 5 Nm), varus-valgus (0 to ± 10 Nm), anterior–posterior translation (0 to ± 100 N). Combined loading tests applied permutations of internal–external rotation moments of − 5, 0, 5 Nm, varus-valgus moments of − 10, 0, 10 Nm, and anterior–posterior drawer forces of − 100, 100 N. During each of these tests, the six degrees-of-freedom loads and movements of the tibiofemoral joint were measured. Patellofemoral joint mechanics were also characterized under quadriceps loading at tibiofemoral flexion angles of 0°, 15°, 30°, 45°, and 60°. At the prescribed tibiofemoral joint pose and orientation, quadriceps loads were applied up to 600 N, and patella kinematics and patellofemoral contact pressures were measured. Anatomical landmarks on femur, tibia, and patella were digitized to establish anatomical coordinate systems. Registration markers on the bones were also probed to provide the opportunity to register mechanical testing coordinate systems with the imaging coordinate system of the same knee. Joint mechanics data were made publicly available as part of a previous effort on data description and dissemination.^9^

### Modeling and Simulation Workflow

**FIGURE 1:**
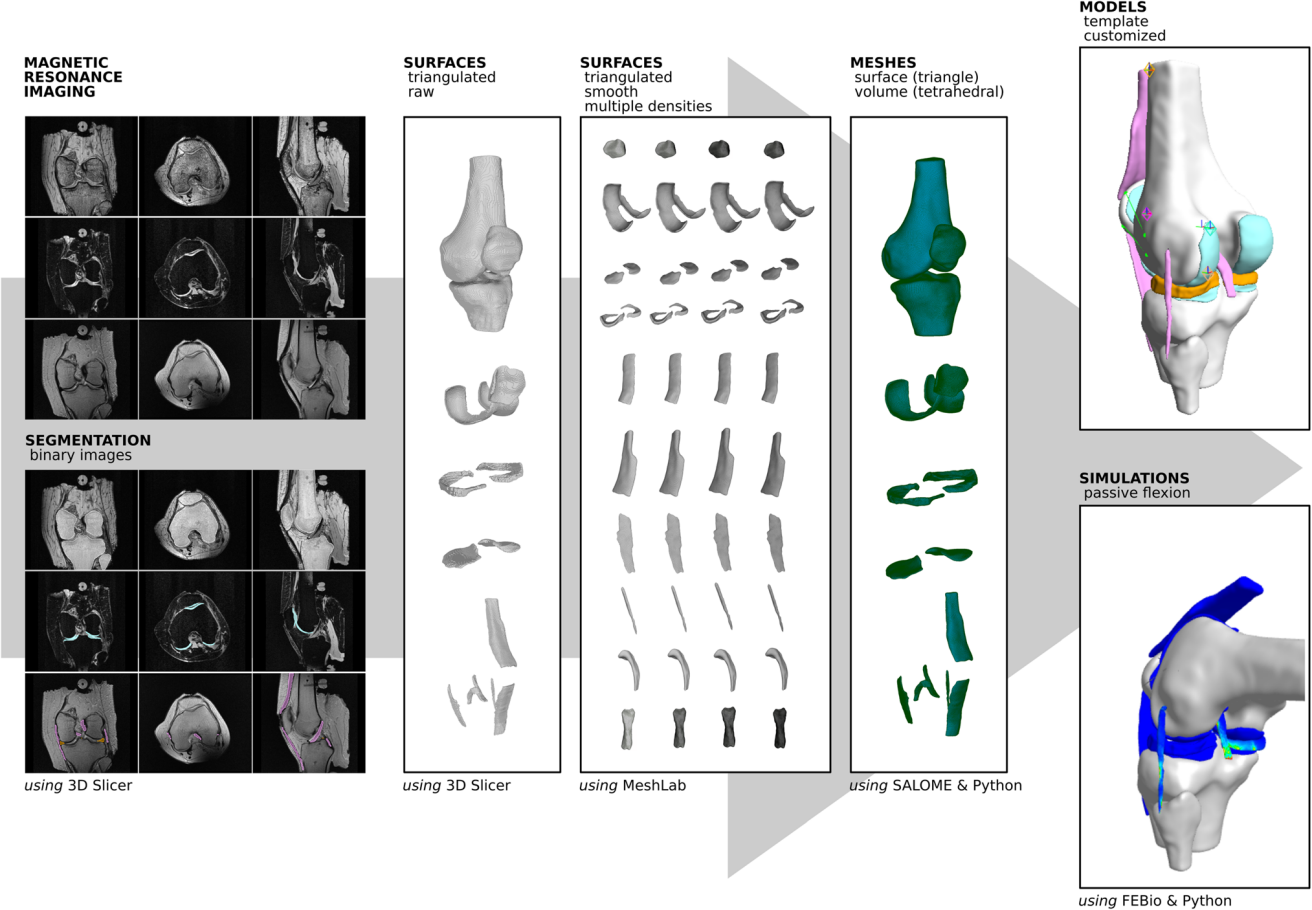
Open Knee(s) model development pipeline to transform MRI of the knees to working models for finite element analysis. Through a sequence of modeling tasks, many digital assets and customizable models were generated along with a simulation demonstration for passive flexion. A variety of free and open source software tools were utilized by the modelers at each step (as indicated).

An end-to-end strategy was implemented to develop the cohort of knee models for finite element analysis (Fig. 1). The approach utilized free and open source software and resulted in a variety of digital assets beyond the final simulation-ready model. The individual steps of the workflow are summarized here. The explicit details, i.e., specifications, of modeling tasks can be found in the supplementary material (Appendix).

#### Image Segmentation

Segmentation for reconstruction of tissue anatomy was done using 3D Slicer.^1,24^ The approach was primarily manual and modelers used common labeling tools such as a brush, pencil etc. to outline, paint or fill in the boundaries of the tissue of interest in a given image volume. Modelers utilized broad and tissue specific segmentation guidance to facilitate the process as documented in the specifications for the Open Knee(s) project (see Appendix). Major tissue structures segmented were the femur, tibia, patella, fibula, medial and lateral menisci, femoral and tibial cartilage, patellar cartilage, medial and lateral collateral ligaments, anterior and posterior cruciate ligaments, patellar ligament and quadriceps tendon. Registration markers that were attached to the femur, tibia and patella were also segmented. These data can be utilized to register knee models to the experiment coordinate systems (see Appendix). The outcomes of the segmentation workflow were tissue labels as binary images (in NIfTI,.nii format) and raw surface meshes (.stl).

#### Surface Geometry Generation

For all tissue structures, smooth and watertight triangulated surface representations were generated interactively, using MeshLab.^10^ A five step heuristically developed procedure facilitated processing of raw surface meshes, which included a staged smoothing approach along with surface reconstruction and resampling. Specifically, a sequence of Laplace smoothing, VCG surface reconstruction, Taubin smoothing, Iso parameterization, Iso parameterization remeshing and Taubin smoothing was used with tissue specific processing parameters—documented as part of geometry generation specifications (see Appendix). The outcome of the geometry generation workflow was surface meshes (.stl) at a variety of resampling levels, i.e., mesh densities. While the initial meshes used in complete knee models (see below) were arbitrarily decided, delivery of multiple surface representations was aimed to support prospective mesh convergence studies.

#### Mesh Generation

Finite element meshes were generated for each tissue structure using surface geometries as inputs. The process was automated using Python scripts and Salome.^43^ For bones, triangulated surface geometries (used in final knee models) were converted to surface meshes with the assumption that these will likely be defined as rigid bodies in finite element analysis. To incorporate into final knee models, tetrahedral volume meshes were generated for all other tissue structures incorporating mesh definitions (nodes, elements) and definitions of regions to facilitate building of models (as node sets, element sets, and surface/face sets). Generation of these sets was automated based on proximity or surface normal analysis of tissue meshes that were defined in a connectivity map as tied, such as ligaments and bone, or interacting, such as contact pairs of cartilage. Following visual inspection and after preliminary simulations, node, element, and face sets were adjusted interactively in Salome (or later in the workflow by editing model files), when and if necessary. Further details of mesh generation can be found in the Appendix. The outcome of mesh generation workflow was meshes of all tissue structures in MED format (version 3.2). Scripts and detailed instructions on how to transform surface geometries (.stl) to finite element mesh (.med) files are also provided as part of the dissemination package.^45^ It should be noted that volumetric meshes of bones can also be generated if they need to be modeled as deformable. Additionally, initial generation of raw geometries (.stl) was at image resolution and provided an upper limit for resolution of the mesh. Thus, full geometric information that can be discerned from images was captured in these raw stl files. Resampling steps coarsen these surfaces. During meshing (surface or volume), linear or quadratic triangular and tetrahedral meshes can be generated. If parameterized by using CAD, sub-image resolution representation of the geometry can be obtained.

#### Model Assembly and Templating

Tissue meshes (with set definitions) were assembled into finite element representations of the knee joints in an automated fashion, using Python scripts and Salome^43^—also part of the dissemination package.^45^ Using a connectivity map and model definition tree as inputs, the scripts generated template models that include place holders for tissue materials, tissue interactions (ties, contacts), and loading and boundary conditions of rigid bodies (bones) (see Appendix). Simulation settings (tolerances, augmentation, etc.) were also defined at this stage. The outcome of template model generation was the descriptions of knee models in FEBio XML markup (.feb, version 2.9.^23^)^33^

#### Model Customization for Simulation-Readiness

**FIGURE 2:**
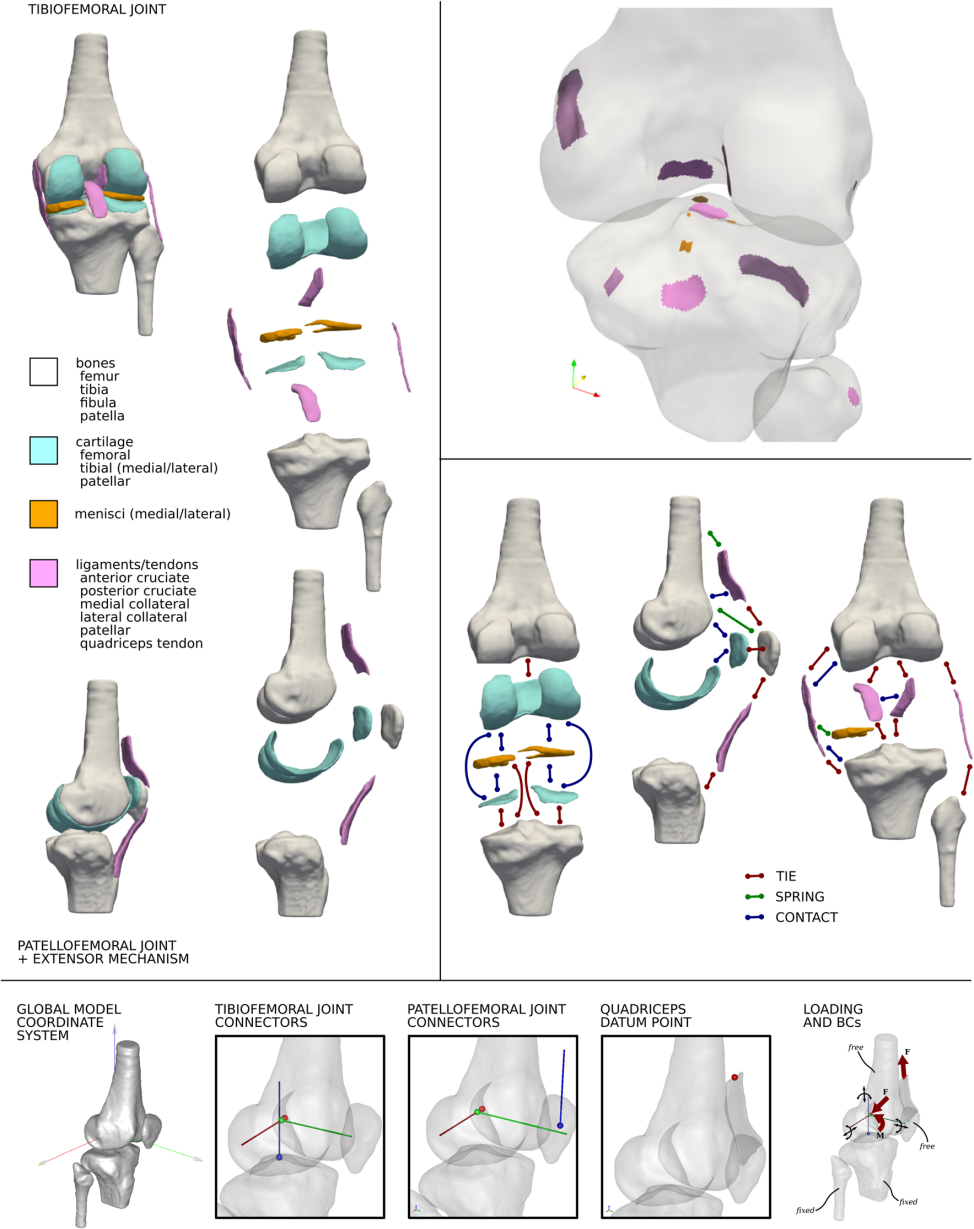
Open Knee(s) model components incorporated in finite element representations of the knee’s passive structures: tissue structures of tibiofemoral and patellofemoral joints (top left), regions within tissues to support model annotation (top right—example shown on femur, tibia, and fibula), interactions between tissue components (middle right—majority are shown), and coordinate systems including joint connectors and loading and boundary conditions (bottom).

Template models were further customized for material definitions, stabilizing components, *in situ* ligament strain, joint coordinate system definitions, loading and boundary conditions, and simulation outputs relevant to computational representation of knee biomechanics (Fig. 2). Bones were rigid; cartilage was nearly incompressible Neo-Hookean; ligaments and tendons were nearly incompressible, transversely isotropic, hyperelastic; and menisci were nearly incompressible, transversely isotropic, hyperelastic. Additional stabilizers were defined. These were: an imaginary rigid body tied to the proximal end of quadriceps tendon and connected to femur with a sliding joint and a linear spring (to constrain the quadriceps tendon) and, discrete elements with force–displacement curve defined as a tension-only linear spring to represent medial and lateral patellofemoral ligaments with insertion and origins based on literature (see Appendix). Also, linear springs were defined connecting each node on the medial collateral ligament in the proximity of the medial meniscus to the nearest node on the side of the medial meniscus. For major ligaments of the tibiofemoral joint (anterior and posterior cruciate ligaments, medial and lateral collateral ligaments) and for those of the extensor mechanism (patellar ligament and quadriceps tendon)* in situ* strain were defined, utilizing the FEBio^23^ Prestrain Plugin^34^ and with values from literature (see Appendix). Many other ligaments such as the anterior intermeniscal ligament and meniscofemoral ligament were not modeled as they were not considered major structures. Medial collateral ligament can be further separated into superficial and deep sections from the imaging data provided previously.^9^ Fiber directions can be defined in the crutiate ligaments to identify bundles as zones within the continuum of the ligament or can be entirely replaced by springs representative of separate bundles.

Based on Grood and Suntay convention,^25^ the femur and tibia coordinate systems were generated using bony landmarks. A kinematics linkage system (and a corresponding joint coordinate system) was implemented by defining cylindrical connectors: a femur fixed axis (for flexion–extension) between femur and an imaginary rigid body, a tibia fixed axis (for internal external-rotation) between tibia and another imaginary rigid body, and a floating axis (for varus-valgus) between imaginary rigid bodies. The patellofemoral joint was represented by a similar kinematic chain, based on anatomical landmarks on the patella, and with patella tilt around a patella-fixed axis and patella rotation around a floating axis. As demonstration case, passive flexion simulation was implemented as model input. Pre-strain application (simulation time 0 to 1) was followed by prescription of flexion up to 90 degrees (simulation time 1 to 2). The tibia and fibula were fixed while the femur and patella were free to move. Flexion was prescribed at the relevant joint connector, where all rotations and translations of other connectors were set free. Apart from default simulation outputs (FEBio), model customization requested ligament stretches (to binary output file,.xplt), and rigid body kinematics-kinetics and joint connector kinematics-kinetics (to text output file,.log). The outcome of model customization was knee models in FEBio XML markup (.feb, version 2.9), ready for passive flexion simulations. All model parameters can be found in the online Appendix and Python scripts to automate customization can be found in the dissemination package.^45^

#### Simulations

Finite element analyses were performed with fully customized models using FEBio (version 2.9), to prescribe* in situ* ligament strains and predict joint movements and tissue stress–strain distributions during passive flexion. This process resulted in binary (.xplt) and text-based (.log) output files. When simulations did not converge, an iterative troubleshooting process helped identify and resolve problems, i.e., by adjusting model parameters, convergence tolerances, alternative solution algorithms and contact formulations (see Appendix). These changes were strictly solver specific. Any updated model with parameters and settings that deviate from previous steps were provided in its final form. The outcome of the simulation step was the delivery of baseline simulation results for passive flexion.

#### Post Processing

A Python script was developed to process simulation results, specifically the FeBio log files, to extract, store, and plot joint kinematics-kinetics. Specifics can be found in the Appendix and the script was included in the dissemination package. This process results in reporting of tibiofemoral and patellofemoral movements and loads in conventional text and graphics formats (.csv,.png). Binary output files generated by FEBio (.xplt) can be further inspected using PostView (version 2.5.0. or above).

#### Additional Utility Scripts

A variety of Python scripts were developed to support prospective use of the models. These range from processing of joint mechanics data (including registration of model and experiment coordinates) to prescription of desired joint kinematics-kinetics as loading and boundary conditions (experimental or otherwise) to compartmental modeling. A sample model calibration strategy and related Python scripts were also provided. We should note that this study focused on demonstration and dissemination of working models and the products of model development workflow. Therefore, further utilization of these scripts is beyond the scope of the study. Nonetheless, their availability will likely facilitate advanced stages of the modeling and simulation lifecycle. Further details describing these processes and scripts can be found in relevant sections of the Appendix. Scripts were also distributed as part of the dissemination package. It should be noted that outputs of all Python scripts were assessed for quality and completeness visually to ensure that the scripts performed as expected.

### Dissemination

All the models and associated digital assets, such as intermediate derivative data and scripts, have been disseminated at a permanent location with unique identification^45^ with permissive licensing to accommodate modifications, reuse, and redistribution for any purpose. Ongoing work extending and using these models can be accessed at Open Knee(s) project website.^46^ The repository is searchable for items such as on donor characteristics. Its organization is summarized in the Appendix.

## Results

**Figure 3:**
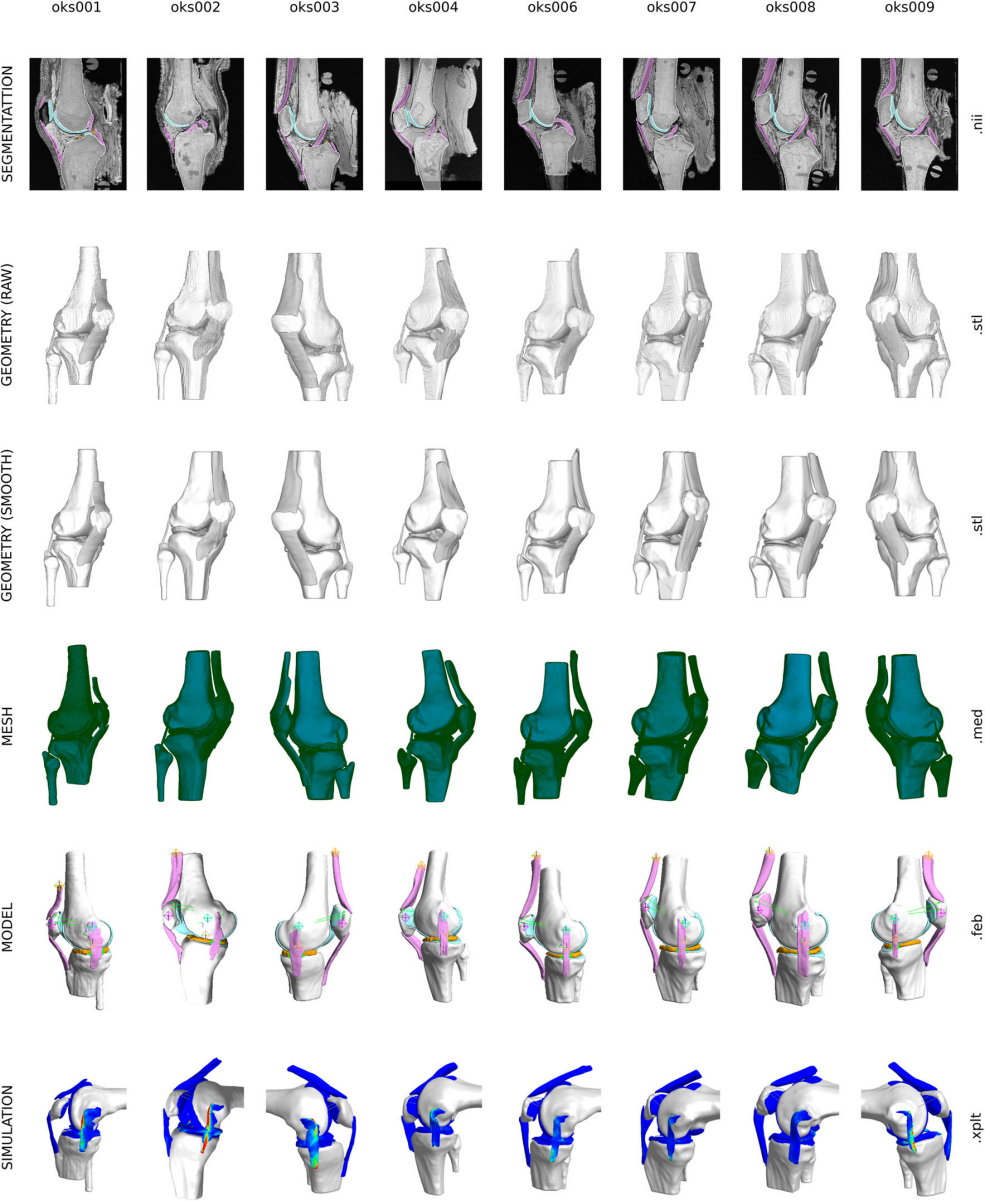
A birds-eye view of Open Knee(s) models and related digital assets. For each knee, more than 100 digital assets were created, representing virtual anatomical and biomechanical representations of the knees and their tissue structures in various forms. Models and digital assets were provided in common file formats (as indicated).

A total of 8 simulation-ready knee models incorporating passive structures of the tibiofemoral joint and the extensor mechanism (including the patellofemoral joint) were built (Fig. 3). A wide array of digital assets were also generated during the transformation of raw imaging data to finite element representations of the knee. For each knee, anatomical models of 16 tissue structures were generated (Fig. 3): femur, tibia, fibula, patella, femoral cartilage, medial and lateral tibial cartilage, patellar cartilage, medial and lateral menisci, anterior and posterior cruciate ligaments, medial and lateral collateral ligaments, patellar ligament and quadriceps tendon. Representation of tissue boundaries and volumes included binary image segmentation labels, raw triangulated surfaces (directly corresponding to segmentation), smooth and watertight triangulated surfaces (ready for meshing), and for all soft tissue structures, volumetric finite element meshes. Average edge lengths for all volume meshes used in the models are provided in the Appendix. For all soft tissue components (cartilage, ligaments, menisci), four levels of surface mesh densities were also created, in support of prospective mesh convergence studies. Meshes that were incorporated into full knee models also had node and element sets and surfaces defined, i.e., as part of annotation of regions that connect or contact with other tissue components (Fig. 2). For example, for the femur, this included all ligament insertion regions, cartilage attachment area, and various contact regions to accommodate ligament wrapping, such as for the medial collateral ligament. Overall, more than 100 digital assets were generated for each knee, beyond specimen-specific imaging and joint mechanics data.^9^

**Figure 4:**
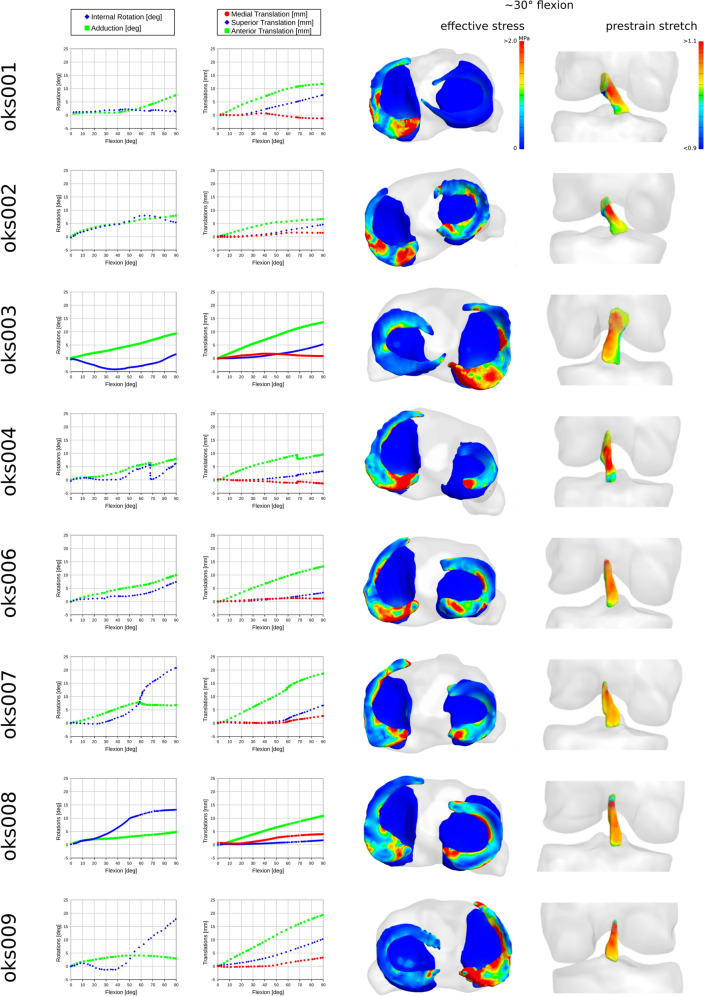
Joint movements and tissue mechanics during passive flexion, obtained by demonstrative finite element analyses of the knee models. From left to right, tibiofemoral joint rotations and translations (joint connector degrees of freedom), effective stress distributions on menisci and tibial cartilage and menisci (at ~ 30° flexion angle), and prestrain stretch (corresponding to total fiber stretch) distributions in anterior cruciate ligament (at ~ 30° flexion angle) are shown for each knee.

To support model modifications and prospective simulations, template models were assembled using tissue meshes, albeit with placeholders for many model components and parameters. This aimed to allow end-users to swap any model definitions easily, such as, constitutive models of tissues. To further demonstrate the value of templating, customization scripts generated models with constitutive models specific to tissue types of the knee, other stabilizers, meaningful coordinate systems, and sample loading and boundary conditions representative of passive flexion (Fig. 3). Passive flexion simulations demonstrated the feasibility to conduct finite element analysis with the knee models. Simulations were usually completed in less than 10 h, providing predictions of joint kinematics-kinetics response and tissue stress–strain distributions for all soft tissue structures. Processed tibiofemoral joint kinematics show the coupled movements of the joint during passive flexion (Fig. 4). Effective stress on tibial cartilage and menisci demonstrates the load sharing on medial and lateral compartments (Fig. 4), even during the unloaded movement of the knee. Similarly, total stretch in the anterior cruciate ligament (stored as prestrain stretch in FEBio) shows its engagement during passive flexion (Fig. 4).

Final models (including sample simulation results) and all digital assets can be found at a permanent location.^45^ Detailed organization of all components for dissemination is described in the Appendix.

## Discussion

To our knowledge, the model and digital assets library generated through the Open Knee(s) initiative is the first of its kind, capturing and disseminating the outcomes of modeling tasks in their entirety for a cohort of knees. The digital assets and models can be traced across all stages of model development and demonstration simulations, all the way back to specimen-specific imaging and joint mechanics data, which were previously disseminated.^9^ Our goal to provide not only easily accessible and usable models but also all the intermediate assets will ensure that if anyone has a suspicion of the misuse of a model, they can rely on the provenance of the models to data and intermediate components for inspection. All the tools utilized for modeling tasks are free and open source, supported by publicly disseminated Python scripts that were developed in house to perform various bridging functions. The cohort may appear small but it is diverse and the distributed specimen-specific modeling assets are extensive.

The majority of publicly available human knee data sets or digital assets are primarily focused on imaging data and subsequently anatomical reconstruction.^22,44,53^ Some investigators working in the area of computational knee biomechanics graciously disseminated data collected on a number of knees,^28,35^ including MRI and computed tomography, joint kinematics-kinetics behavior, and models for finite element analysis. However, the raw data from these studies were not comprehensive, models were simplified, and intermediate outcomes of modeling tasks were not available completely. This situation may limit the provenance and usability of the data set and thus it was a motivation for our extensive curation and dissemination. An important contribution of this study is the delivery of detailed specifications on how each stage in the workflow was accomplished so that the users can comprehend and reproduce modeling tasks themselves (see Appendix). This level of transparency was aimed at promoting reproducibility and credibility^16,20^ but also identifies areas that may benefit from automation. For example, automated segmentation is an obvious need for any modeling strategy utilizing anatomy and can be achieved depending on tissue types and imaging modality. Nonetheless, transforming data into a working model necessitates many other time consuming and interactive tasks.^19,41^ If automated, they may accelerate the delivery of models and interpretation of simulation results. We developed and disseminated an array of Python scripts to streamline meshing, annotation of mesh regions, model assembly, templating, customization, and post-processing to reduce the need for manual intervention. Our first generation Open Knee(s) model^15^ and its derivative data has been extensively used in numerous studies since it was first made publicly accessible. With this new extensive database for a number of specimens, we aim to reach an even wider group of researchers and clinicians. Availability of these digital assets will provide an opportunity to pursue the research goals more expeditiously to those researchers, who may not have access to extensive resources, or may not have the capacity and time required to generate them. Potential users can directly use the simulation-ready models to conduct *in silico* studies, with or without model modification, as we have demonstrated with passive flexion simulations. They may extract compartmental models for tissue focused finite element analysis, such as, for ligaments. Since the derivative data are available, users can also choose any one of the outputs of the modeling tasks (segmentation, geometry generation etc.) to start their workflow, reproduce model development steps, change model fidelity, or utilize them for purposes other than finite element analysis, i.e., anatomical model templating. Accessibility of all these assets also permits the users to perform a thorough quality check.^17^ Association of specimen-specific joint mechanics and imaging data establishes grounding at a specimen level and many customization opportunities starting with the data. The database of knee models and related digital assets is searchable, supporting discoverability by queries across specimens as well as within the specimens. We should note that the research team has also curated tissue samples (cartilage, menisci, ligament sections) from the same specimens of the Open Knee(s) cohort, as a supporting physical repository to allow tissue level mechanical and/or histological characterization. An extensive cartilage mechanical testing for one of the knee specimens (oks003) was already performed and disseminated.^7^

Our efforts aimed to diligently plan, execute, and capture the whole model development process. Nonetheless, some limitations remain. Density of meshes to include in the final models were chosen heuristically. However, several surface meshes were provided to let the users swap the tissue meshes and perform systematic mesh convergence analyses. Manual nature of segmentation is an error prone process due to the knowledge and experience of the operator, image quality, available tools and tool preferences of the operator. Since the segmentation burden was high, several operators with varying degrees of experience participated in the process. All segmentations were visually assessed but some details may still have not been captured. Segmentation of ligaments is particularly challenging and may need to be evaluated and redone by the end-users. That being said, modifications in segmentations and surface geometries can be incorporated into the models easily, using the Python scripts to replace mesh components. We should also note manual adjustments to node sets, particularly those that were assigned to rigid bodies as tie constraints (ligament insertions, for example). These adjustments were needed either after visual inspection, to capture a more realistic representation of the insertion footprint, or to alleviate convergence issues caused by elements that were deforming severely. Simulation tolerances (such as displacement, energy, force residuals), solution settings (iteration parameters), and augmentation (for satisfaction of contact iterations and other constraints) were adjusted from default FEBio settings, to ensure convergence and to reduce computational cost. For some material model applications, additional steps may be necessary, such as meshing differently or explicit assignment of material directions, when and if a user would prefer to change the material model. We should note that our transversely isotropic model for the meniscus already provides an example of how explicit directions can be assigned for each element of a tetrahedral mesh (see associated Python scripts). This readily embedded information can be utilized to define fiber directions in fiber-reinforced models.

A comprehensive assessment of reproducibility of manual steps and its impact on model predictions was not performed. A representative assessment of potential inter-user variability in segmentation was performed previously^9^ where three users with varying level of experiences conducted segmentation of the same structure. The variations were mostly within sub image resolution. However, all the specifications were followed with the acknowledgement that there is a certain ‘art” involved in modeling and simulation processes that may have introduced discrepancies^41^ due to user decision and experience.

Simulation demonstrations confirmed the readiness of the knee models to conduct finite element analysis (Fig. 4). Passive flexion is a characteristic movement of the knee joint guided by articular contact and ligament restraints. The i*n silico* knee cohort exhibited coupled movements, such as internal–external rotation as the virtual knee was flexed, as expected. Stresses at contact regions and ligament deformations (Fig. 4), demonstrate their utility in guiding knee movement. We should emphasize that we did not conduct any validation in regard to capturing the specimen-specific realism of these movement patterns. Verification and validation tasks and acceptable tolerances for establishing credibility are tightly coupled to the “context of use” of a model.^2,20^ Thus, we did not want to label our models as “valid” without knowing what prospective users’ context of use may be. Availability of specimen-specific experimental joint mechanics data provides any end-user the opportunity to conduct validation studies that resonates with their use case’s intended loading scenarios. A handful of Python scripts were provided to facilitate processing of joint mechanics and registration marker data. Availability of various mesh densities for tissue structures also provides the opportunity for context relevant solution verification. We want to emphasize that there is a risk of usage without proper understanding of the model development process and the associated skills, knowledge and challenges. The availability of all the components would allow the users and eventually their audience to assess the quality of their work based upon these data.

In a multi-institute sister project on reproducibility and art of knee modeling,^19^ we were able to extensively calibrate and benchmark one of the knee models (oks003; see KNEEHUB project site for relevant materials^47^). A mesh convergence analysis was completed, material properties were adjusted for ligaments, cartilage, and menisci and,* in situ* ligament strains were optimized to partially fit joint mechanics data. The model was registered to the experimental coordinate system and experimental loads were applied to the model. Extensive post calibration simulations were run and benchmarking was performed using joint laxity and combined joint loading data. This experience demonstrated how the models that we disseminate can be further advanced. Reporting of this sample calibration and benchmarking study is beyond the scope of this manuscript. Nonetheless, to empower the users of Open Knee(s), relevant documentation of this sample workflow and supporting Python scripts were shared in the Appendix and the dissemination package.^45^

With all limitations acknowledged, the dataset establishes a comprehensive virtual cohort—with all digital components generated throughout model development, as a platform to facilitate virtual experiments on knee biomechanics. Completeness of the virtual assets, their association to specimen-specific experiments, and the transparency and comprehensive detail of modeling workflows will ensure their credible and expeditious reuse. Thus, this study sets a strong example of FAIR practices^50^ in computational biomechanics. This dissemination represents a point-in-time snapshot for Open Knee(s). That being said, the database is anticipated to grow, either by advancement of models for the existing cohort or by acquisition and curation of data and development of models for additional knees.

## Supplementary Information

Below is the link to the electronic supplementary material (Appendix).

Supplementary file1 (PDF 2432 kb)
