# Supplementary material for: Open Knee(s): A Free and Open Source Library of Specimen-Specific Models and Related Digital Assets for Finite Element Analysis of the Knee Joint
Source: Ann Biomed Eng. 2022 Sep 14;51(1):10–23. doi: 10.1007/s10439-022-03074-0 (PMC9832097; doi:10.1007/s10439-022-03074-0)
Supplement: Supplementary file 1 — Supplementary file1 (PDF 2432 kb) [file 10439_2022_3074_MOESM1_ESM.pdf]

Open Knee(s): A free and open source library of specimen-specific models and related digital assets for finite element analysis of the knee joint

Appendix

Snehal Chokhandre, Ariel Schwartz, Ellen Klonowski, Benjamin Landis, Ahmet Erdemir

Department of Biomedical Engineering, Lerner Research Institute, Cleveland Clinic, Cleveland, OH, United States.

Table of contents

A1. Modeling and simulation workflow – Model development 2
Image Segmentation 2
Geometry Generation 6
Volume Mesh Generation 11
Template Model Generation 15
Model Customization 17
Simulation 28
Post-Processing 29
A2. Experimental data processing 30
Registration for Specimen-Specific Calibration 30
Specimen-Specific Kinematics-Kinetics Data Processing 32
A3. Model customization for application of kinematics-kinetics (with or without experimental data) 34
A4. Model calibration procedure 35
Mesh Convergence 35
Confirmation of Material Properties 39
Calibration of In Situ Ligament Strains 42
A5. Edge lengths calculated for all meshes used in the models 45
A6. Model specific measures for convergence 46
A7. Database folder structure 47
A8. Python scripts and usage 48
References 52

A1. Modeling and simulation workflow – Model development

Image Segmentation

Target outcome Volumetric reconstruction of the tissues of interest, specifically the definition of the tissue boundaries, as binary image volumes (.nii; NIfTI)[8] and raw (without smoothing) triangulated surface representations (.stl)[29] in the same coordinate system as imaging data. Tissues include bones – femur, tibia, fibula, patella; cartilage – femoral, tibial (medial & lateral), patellar; menisci – medial & lateral, ligaments – anterior/posterior cruciate, medial/lateral collateral, patellar; tendons – quadriceps.

Software requirements 3D Slicer**.** 3D Slicer[1] is a free, open source software package for visualization and image analysis (free and open source, BSD style, licensing, see http://www.slicer.org). The latest version at the time (Slicer 4.8) was used to access more tools and features.

Input Set(s) of MRI from Open Knee(s) in NIfTI format (https://nifti.nimh.nih.gov/)[8] (all in the same coordinate system):

1. General Purpose MRI (3D T1-weighted without fat suppression, isotropic, 0.5 mm resolution)
2. Cartilage MRI (3D T1-weighted with fat suppression, 0.35 mm sagittal plane resolution, 0.7 mm out of plane resolution)
3. Connective tissue MRI (proton density, turbo spin echo in sagittal, axial, and coronal planes, 0.35 mm in plane and 2.8 mm out of plane resolution)

Segmentation algorithms The segmentation approach was primarily manual, requiring the modeler to use common labeling tools, such as, brush, pencil, etc. to paint or fill in the tissue region of interest in image volume. Depending on the tissue, multiple image volumes were used to confirm tissue boundaries or assist the tissue volume generation. Several 3D Slicer tools were also used during the segmentation process to assist gross segmentation before manual touch up. These are described in the following.

1. GrowCut Segmentation

From 3D Slicer documentation[1]:

"GrowCut Segmentation is a competitive region growing algorithm using cellular automata. The algorithm works by using a set of user input scribbles for foreground and background. For N-class segmentation, the algorithm requires a set of scribbles corresponding the N classes and a scribble for a don't care class."

In this workflow, GrowCut was used primarily for gross segmentation of large tissue volumes.

2. Label Map Smoothing

From 3D Slicer documentation[1]:

"This filter smooths a binary label map. With a label map as input, this filter runs an anti-aliasing algorithm followed by a Gaussian smoothing algorithm. The output is a smoothed label map."

In the smoothing parameters outlined below, Sigma (Gaussian smoothing parameter) was chosen based on image resolution. For example, for cartilage MRI with resolution = 0.35 x 0.35 x 0.7 mm, sigma was set to 0.7. It should be noted that label map smoothing was used to assist segmentation as an intermediate step and should not be confused with preparation and smoothing of raw geometry for meshing (see section on Geometry Generation).

3. Joint Smoothing

From 3D Slicer documentation[1]:

"This will ensure that all resulting models fit together smoothly, like jigsaw puzzle pieces. Otherwise the models will be smoothed independently and may overlap."

It should be noted that joint smoothing was used to assist segmentation as an intermediate step and should not be confused with preparation and smoothing of raw geometry for meshing (see section on Geometry Generation).

Tissue-Specific Segmentation Procedures Below are the guidelines for segmentation procedures based on tissue types. It is important to use the specified input MRI, and locate the boundary as described for each tissue. However, the segmentation procedures described are only suggestions, and one may choose to highlight the anatomy using whichever tool they find most effective and efficient for their manual workflow. Once all tissues were segmented, the label maps were saved as NIfTI files (.nii)[8] and converted to raw triangulated surfaces (.stl[29], in units

mm) (without any further smoothing procedures). Slicer provides output options to accommodate these formats.

Tissue/Object	Input MRI	Boundary Definition*	GrowCut Segmentation#	Manual Segmentation	Label Map Smoothing#	Joint Smoothing#
**Registration Markers$**<br><br>femoral (x3)<br><br>tibial (x3)<br><br>patellar (x3)	General purpose MRI	Markers appeared bright in the MRI – the outer edge of the bright region defines the boundary.	yes	yes<br><br>(fill in screw holes/ bubbles)		
**Bones**<br><br>femur<br><br>tibia<br><br>fibula<br><br>patella	Cartilage MRI	Cortical bone appeared black in MRI – the outer edge of black region defines the bone surface.	yes		yes<br><br>sigma=0.7	
**Cartilage**<br>femoral<br><br>tibial (medial)<br><br>tibial (lateral)<br><br>patellar	Cartilage MRI	Use bone boundary to help define cartilage boundary at bone interface. Cartilage appeared bright white – the outer edge defines articulating surface.			yes<br><br>sigma=0.7	yes<br><br>(with bones)
**Menisci**	Cartilage MRI	Use cartilage boundary to		yes	yes	yes

Tissue/Object	Input MRI	Boundary Definition*	GrowCut Segmentation#	Manual Segmentation	Label Map Smoothing#	Joint Smoothing#
medial<br><br>lateral		help define meniscus boundary. Meniscus appeared darker.			sigma=0.7	(with cartilage)
**Ligaments**<br><br>**Tendons**<br><br>ACL<br><br>PCL<br><br>patellar ligament<br><br>quadriceps tendon	Connective tissue MRI<br><br>(for boundary)<br><br>General purpose MRI<br><br>(for resolution)	Use bone boundary to help define connective tissue boundary at insertion and origin sites. Ligaments and tendons appeared dark.	yes<br><br>Phase 1:<br><br>using sagittal connective tissue MRI	yes<br><br>Phase 2:<br><br>using general purpose MRI, overlay labelmap from Phase 1 (as foreground layer) and trace boundary&		yes<br><br>(with bones)
**Ligaments**<br><br>LCL<br><br>MCL	General purpose MRI<br><br>(for boundary)<br><br>Connective tissue MRI<br><br>(for confirmation)	Use bone boundary to help define ligaments in coronal plane. Ligaments appeared dark.		yes	yes<br><br>sigma=0.5	

ACL: anterior cruciate ligament. PCL: posterior cruciate ligament. LCL: lateral collateral ligament. MCL: medial collateral ligament.

#Always check the image slices manually after using GrowCut Segmentation or Label Map and Joint Smoothing, and perform touch ups as needed to better define the boundaries.

$While segmentation of reference markers are not needed to obtain an initial model, they are included in the workflow with the anticipation of their utility in upcoming modeling and simulation stages.

&Alternatively, one can display the connective tissue MRIs and the general purpose OR cartilage MRI in different windows. When linked, Slicer uses interpolation for coupled viewing of the image sets that are already spatially aligned. In return, one can do the segmentation on interpolated connective tissue MRIs using the general purpose OR cartilage MRI as the master volume for segmentation. This allows high resolution segmentation volume from images with lower resolution directly.

Geometry Generation

Target outcome Geometric reconstruction of the tissues of interest as smooth and watertight triangulated surface representations (.stl)[29] obtained from and in the same coordinate system as raw geometry; ready for volumetric meshing. Tissues include bones – femur, tibia, fibula, patella; cartilage – femoral, tibial (medial & lateral), patellar; menisci – medial & lateral, ligaments – anterior/posterior cruciate, medial/lateral collateral, patellar; tendons – quadriceps.

Software requirements MeshLab. MeshLab[7] is an open source, portable, and extensible system for processing and editing of unstructured 3D triangular meshes (free and open source GPL license, see http://www.meshlab.net/). The latest version of MeshLab (MeshLab 2016.12) was used.

Input Raw triangulated surface representations of tissues of interest (without filtering and smoothing) in .stl[29] format in image coordinate system.

Surface processing procedures A 5-step procedure (LVTIT, see below for descriptions) was used to process raw triangulated surface meshes. The process included staged smoothing approaches interleaved with surface reconstruction and resampling. The procedures aimed to generate uniform and watertight surface meshes that were smooth, volume-preserving and visually maintaining the geometrical shape of the tissues.

Smoothing Algorithms This section provides brief descriptions of the MeshLab[7] algorithms that were used during processing of triangulated surface meshes.

1. <u>Laplacian Smoothing [L]</u>

For each vertex in the mesh, a new position is chosen based on average position with nearest vertex (as described in built-in documentation in MeshLab[7]). The "Smoothing steps" parameter is the number of times the process is repeated.

2. <u>Surface Reconstruction: VCG [V]</u>

From built-in documentation in MeshLab[7]:

> "Surface reconstruction algorithm that have been used for a long time inside the ISTI-Visual Computer Lab. It is mostly a variant of the Curless at al. e.g. a volumetric approach with some original weighting schemes, a different expansion rule, and another approach to hole filling through volume dilation/relaxations."

"Voxel Side" values should be informed in relation to original image resolution from which raw surfaces are obtained; e.g., for cartilage images with a resolution of 0.35 x 0.35 x 0.7 mm, use a world unit of 0.35, 0.5, or 0.7 mm.

3. <u>Taubin Smoothing [T]</u>

From built-in documentation in MeshLab[7]:

> "The λ-μ Taubin smoothing, it makes two steps of smoothing, forth and back, for each iteration. Based on Gabriel Taubin, A signal processing approach to fair surface design, Siggraph 1995"

4. <u>Iso Parameterization [I]</u>

Stage 1: Iso Parameterization

From built-in documentation in MeshLab[7]:

> "The Filter builds the abstract Isoparameterization of a two-manifold triangular mesh. An adaptively chosen abstract domain of the parameterization is built. For more details see:

> Pietroni, Tarini, and Cignoni, 'Almost isometric mesh parameterization through abstract domains' IEEE Transaction of Visualization and Computer Graphics 2010"

Stage 2: Iso Parameterization Remeshing

From built-in documentation in MeshLab[7]:

> "Remeshing based on Abstract Isoparameterization, each triangle of the domain is recursively subdivided. For more details see Pietroni, Tarini, and Cignoni, 'Almost isometric mesh parameterization through abstract domains' IEEE Transaction of Visualization and Computer Graphics 2010"

"Sampling Rate" determines the number of subdivisions, aka. density of the surface mesh.

Tissue specific processing parameters The table below specifies the parameters used in the surface mesh processing for each tissue for the surface meshes that were used in the generation of provided models. These values were considered a starting point and the process was performed in the order provided (if '-', skip step). For parameters that were not specified MeshLab defaults were used. The outcome of surface mesh processing needed to be checked (visually and when possible, quantitatively) to ensure that there was no significant loss of geometric features or volumes. If necessary, surface repairing was performed, i.e. using other MeshLab[7] tools, to ensure a manifold and watertight surface; sometimes faces needed to be re-oriented such that surface normals all pointed outward. The final surface representation of the tissue was (.stl[29], in units mm). It should be noted that these parameters were developed using one specimen and needed some specimen specific adjustments for the remaining specimens. Due to manual application of these parameters, some decisions made by the modelers may not be documented (several modelers with varying experience were involved in this process). File names typically consisted of some sequence of the smoothing step to provide an indication of which steps were used for a given surface, however exact details of parameters were not documented as the decisions were made based on visual inspections of how the surfaces looked after each step and whether any changes were required to create water tight meshes. Oks003 surface meshes were created using the same parameters as the additional surface meshes provided later in the document.

	1. Laplacian Smoothing (Smoothing Steps)	2. VCG Surface Reconstruction (Voxel Size, mm)	3. Taubin Smoothing (Lambda, mu)	4. Iso Parameterization (Sampling Rate)*	5. Taubin Smoothing (Lambda, mu)
**Registration Markers**	-	-	-	-	-
**Femur, Tibia**	20	0.7	0.5,-0.53	10	0.5,-0.53
**Patella**	20	0.5	0.5,-0.53	7	0.5,-0.53
**Fibula**	20	0.5	0.5,-0.53	5	0.5,-0.53
**Femoral Cartilage**	20	0.35	0.5,-0.53	10	0.5,-0.53
**Tibial Cartilage**	20	0.35	0.5,-0.53	10	0.5,-0.53
**Patellar Cartilage**	20	0.35	0.5,-0.53	10	0.5,-0.53
**Menisci**	20	-	-	10	0.5,-0.53
**ACL,PCL**	4	0.35	0.5,-0.53	5	0.5,-0.53
**Patellar Ligament**	20	0.35	0.5,-0.53	10	0.5,-0.53
**Quadriceps Tendon**	20	0.35	0.5,-0.53	10	0.5,-0.53
**MCL**	4	0.35	-	7	0.5,-0.53
**LCL**	2	0.35	-	5	0.5,-0.53

ACL: anterior cruciate ligament. PCL: posterior cruciate ligament. LCL: lateral collateral ligament. MCL: medial collateral ligament.

Additional surface geometries

To facilitate mesh convergence studies, additional multiple surfaces for each component were generated. Care was taken to use the following parameters consistently. An effort was made to document any deviations. However, it should be noted that the stochastic nature of software used may give different results when using the same parameters. For specimen oks003, additional surface meshes were created from the smoothed surface meshes created for the model.

	**1. Laplacian Smoothing (Smoothing Steps)**	**2. VCG Surface Reconstruction (Voxel Size, mm)**	**3. Taubin Smoothing (Lambda, mu)**	**4. Iso Parameterization (Sampling Rate)***	**5. Taubin Smoothing (Lambda, mu)**
**Registration Markers**	-	-	-	-	-
**Femur, Tibia**	20	0.7	0.5,-0.53	10	0.5,-0.53
**Patella**	20	0.5	0.5,-0.53	7	0.5,-0.53
**Fibula**	20	0.5	0.5,-0.53	5	0.5,-0.53
**Femoral Cartilage**	15	0.28	0.5,-0.53	8,10,15,20	0.5,-0.53
**Tibial Cartilage**	20	0.35	0.5,-0.53	6,8,10,15	0.5,-0.53
**Patellar Cartilage**	20	0.35	0.5,-0.53	6,8,10,15	0.5,-0.53
**Menisci**	5	0.3	0.5,-0.53	4,6,8,10	0.5,-0.53
**ACL**	2	0.35	0.5,-0.53	4,6,8,10	0.5,-0.53
**PCL**	4	0.35	0.5,-0.53	3,4,6,8	0.5,-0.53
**Patellar Ligament**	20	0.35	0.5,-0.53	4,6,8,10	0.5,-0.53
**Quadriceps Tendon**	20	0.35	0.5,-0.53	4,6,8,10	0.5,-0.53

	1. Laplacian Smoothing<br><br>(Smoothing Steps)	2. VCG Surface Reconstruction<br><br>(Voxel Size, mm)	3. Taubin Smoothing<br><br>(Lambda, mu)	4. Iso Parameterization<br><br>(Sampling Rate)*	5. Taubin Smoothing<br><br>(Lambda, mu)
**MCL**	4	0.35	-	5,7,9,11	0.5,-0.53
**LCL**	2	0.35	-	3,4,6,8	0.5,-0.53

ACL: anterior cruciate ligament. PCL: posterior cruciate ligament. LCL: lateral collateral ligament. MCL: medial collateral ligament. TBC-L: Tibia cartilage – lateral.

Volume Mesh Generation

Target outcome Mesh definitions (nodes, elements, surfaces; node, element, face sets), template constitutive models (rigid – bones; deformable – other tissue). Outputs included tetrahedral volume meshes (cartilage, menisci, ligaments, tendon) in binary format (.med)[24] with node, face, element sets to facilitate model assembly, material property definitions, and assignment of tissue interactions.

Software requirements SALOME**.** SALOME[26] is an open-source software that provides a generic platform for pre- (cad, meshing) and post-processing for numerical simulation (free and open source LGPL license, see http://www.salome-platform.org/) SALOME[26] includes built-in scripting functionality using Python[32], which was required to utilize Python scripts for mesh generation and annotation, and model assembly. SALOME[26] 7.8.0 was used to support in-house Python[32] scripts.

StlToMed.py. In-house Python[32] script to generate meshes (surface or volume) and node, element, face sets using an XML based input file that points to .stl[29] files, e.g., tissue geometries, and their connectivity. To be used with SALOME's built-in Python installation; developed using SALOME[26] 7.8.0, source code available at https://simtk.org/plugins/datashare/?group_id=485# (OKS_model) – DOI pending.

ConnectivityXML.py. Python[32] utility script to read an XML file that points to .stl[29] files and their connectivity for model assembly. Source code available at https://simtk.org/plugins/datashare/?group_id=485# (OKS_model) – DOI pending.

.

Input Geometric reconstruction of tissues of interest – smooth and watertight triangulated surface representations (.stl)[29], all in the same coordinate system, e.g., image coordinate system. Also a description of model components and their interactions with each other, essentially a model definition tree, as an input file to Python[32] scripts. This file included references to all tissue components that were part of the model: bones – femur, tibia, fibula, patella; cartilage – femoral, tibial (medial & lateral), patellar; menisci – medial & lateral, ligaments – anterior/posterior cruciate, medial/lateral collateral, patellar; tendons – quadriceps. Each tissue entry pointed to the file location of triangulated surface representation of the tissue boundary. Each tissue was referred to as “rigid” (bones) or “elastic” (other tissues) to generate relevant place holders for material definitions and loading and boundary conditions during model assembly. In addition, interactions between tissues were defined as “Tie” or “Contact” to define kinematic constraints or contact, respectively, between opposing regions of tissues. Determination of surfaces, e.g. face sets, and/or node sets for these constraints was based on geometric principles relating to the pair of meshes based on:

- Proximity – find all the nodes on part 1 that are within a prescribed distance (related to the element size) of any node on part 2.
- Normals – select faces on part 1 that have normal vectors that point toward the barycenter of part 2. This can also be limited by the proximity condition if desired.
- Contains – one mesh contains the other, select elements that have normal vectors that point inward or outward.
- All – all the surfaces.

This analysis was automated using the Python[32] scripts with SALOME[26] during mesh generation and annotation. The following table specifies all the tie constraints and contacts surfaces between tissues, and which geometrical principles should be used to relate them.

	**FM**	**TB**	**FB**	**PT**	**FC**	**TLC**	**TMC**	**PC**	**MM**	**LM**	**ACL**	**PCL**	**MCL**	**LCL**	**PL**	**QT**
**FM**					T:p						T:p	T:p	C:n<br>T:p	C:n<br>T:p		C:n
**TB**						T:p	T:p		T:p	T:p	T:p	T:p	C:n<br>T:p		T:p	

**FB**														T:p		
**PT**								T:p							T:p	T:p
**FC**	T:p					C:n	C:n	C:n	C:n	C:n						C:a
**TLC**		T:p			C:a					C:n						
**TMC**		T:p			C:a				C:n							
**PC**				T:p	C:n											
**MM**		T:p			C:n		C:n						T:p			
**LM**		T:p			C:n	C:n										
**ACL**	T:p	T:p										C:a				
**PCL**	T:p	T:p									C:p					
**MCL**	C:n<br>T:p	C:a<br>T:p							T:p							
**LCL**	C:n<br>T:p		T:p													
**PL**	T:p			T:p												
**QT**	C:n			T:p	C:n											

FM: femur. TB: tibia. FB: fibula. PT: patella. FC: femoral cartilage. TLC: tibial lateral cartilage. TMC: tibial medial cartilage. PC: patellar cartilage. LM: lateral meniscus. MM: medical meniscus. ACL: anterior cruciate ligament. PCL: posterior cruciate ligament. MCL: medial collateral ligament. LCL: lateral collateral ligament. PL: patellar ligament. QT: quadriceps tendon. – C: Contact. T:Tie. p: proximity, n: normals. c: contains. a: all.

Mesh generation and annotation Use of StlToMed.py with SALOME's built-in Python[32] and the previously described connectivity file resulted in meshes of the tissue components along with node, element and surface definitions and node, element, face sets (for material property assignment, tie and contact definitions, and loading and boundary assignment). The script was

automated, resulting in mesh files (in .med[24] format and in units of the input files) for each tissue component. The script starts with generating triangular surface meshes (for rigid objects – bones) and tetrahedral (four node) volume meshes (for elastic objects – other tissues) using geometric reconstructions of the tissue (.stl)[29]. Surface mesh discretization for rigid objects is identical to that of the input .stl[29] file. Surface discretization of elastic objects is identical to that of the input .stl[29] file. Volume meshing utilizes SALOME's[26] interface to NETGEN 3D mesh[19] generator with the following settings (see SALOME NETGEN documentation for more details[25]):

**Name**	**Definition**	**Value**
Max Size	maximum linear dimensions for mesh cells.	Equal to the maximum linear dimension of the input STL[29]
Min Size	minimum linear dimensions for mesh cells.	Equal to the minimum linear dimension of the input STL[29]
Fineness	ranging from *Very Coarse* to *Very Fine* allows to set the level of meshing detailization.	Level 4. (If this fails, use Level 3)
Optimize	the algorithm modifies initially created mesh in order to improve quality of elements. Optimization process is rather time consuming comparing to creation of initial mesh.	On

The script automatically generated an element set including all elements within the tissue, which was later used to assign material properties. Node and surface regions for tie and contact definitions were determined based on previously noted "Proximity", "Normals", or "All" settings denoted in the connectivity file. For "Proximity" a multiplier value of 1.0 was used to scale the approximate characteristic length of the surface mesh to determine the proximity threshold. For "Normals" setting a multiplier was not used.

Meshes of tissues were visually inspected in SALOME[26]. This inspection facilitated confirmation of appropriate definitions of node and surface regions to prescribe tissue connectivity, e.g., ligament insertion and origin sites, and contact, e.g. between cartilage. If necessary these sets were interactively edited in SALOME[26] by adding/removing nodes, faces, etc. from groups.

Template Model Generation

Target outcome Template model for finite element analysis in FEBio[11] format (.feb[13], XML[20] based text file) including mesh definitions (nodes, elements, surfaces; node, element, face sets), template constitutive models (rigid – bones; deformable – other tissue), template interactions between tissue (tie constraints, contact), and template loading and boundary conditions (for rigid objects); created from geometric reconstruction of tissues.

Software requirements SALOME**.** SALOME[26] is an open-source software that provides a generic platform for pre- (cad, meshing) and post-processing for numerical simulation (free and open source LGPL license, see http://www.salome-platform.org/). SALOME[26] includes built-in scripting functionality using Python[32], which was required to utilize Python scripts for mesh generation and annotation, and model assembly. SALOME 7.8.0[26] was used to support in-house Python[32] scripts.

MedToFebio.py. In-house script to generate a template FEBio[13] model using XML[20] based connectivity file and meshes, e.g. output of StlToMed.py. To be used with SALOME's[26] built-in Python installation; developed using SALOME 7.8.0[26], supports FEBio[13] file format version 2.5, source code available at https://simtk.org/plugins/datashare/?group_id=485# (OKS_model).

ConnectivityXML.py. Python utility script to read an XML[20] file that points to .stl[29] files and their connectivity for model assembly. Source code available at https://simtk.org/plugins/datashare/?group_id=485# (OKS_model).

Input tetrahedral volume meshes (cartilage, menisci, ligaments, tendon) in binary format (.med)[24] with node, face, element sets to facilitate model assembly, material property definitions, and assignment of tissue interactions.

Model Assembly Use of MedToFebio.py with SALOME's[26] built-in Python[32], the previously described connectivity file, and mesh files (outcome of mesh generation and annotation step) resulted in a template FEBio[13] model file (.feb[13], version 2.5) for finite element analysis. The script is automated and resulted in the following contents written in the model file:

- Node definitions – list of the node coordinates for each tissue
- Element definitions – list of node connections to define the elements for each tissue
- Node sets, face sets, element sets, surface definitions – groups of nodes, elements, faces labeled to be used in tie and contact pairs
- Material definitions

- For rigid bodies (bones) only a place holder density is assigned (1e-9 tonnes/mm^3 – consistent with spatial units of mm)
- For elastic bodies (other tissues) a place holder density is assigned as 1e-9 tonnes/mm^3 and a Neo-Hookean material is defined using FEBio material type Mooney-Rivlin (uncoupled)[12] and setting c1 = 0.1 MPa; c2 = 0 MPa; k = 100 MPa (consistent with spatial units of mm) as a place holder.

- Surface pairs – to define connections (ties) or interactions between the surfaces, specifies a "master" surface, and "slave" surface by using the following criteria:
    - The surface on a rigid body should be the master surface.
    - The larger of the two surfaces should act as the master surface.
    - If the surfaces are of comparable size, the surface on the stiffer body should act as the master surface.
    - If the surfaces are of comparable size and stiffness, the surface with the coarser mesh should act as the master surface.
- Tied interfaces – "tied" type contact between deformable surface pairs (penalty = 10)
- Contact interactions – "facet-to-facet-sliding" type contact (called "sliding-facet-on-facet" in recent versions of FEBio) between surface pairs (frictionless, laugon = 1, penalty = 1, auto_penalty = 1, maxaug = 10)
- Loading and boundary conditions
    - Assignment of nodes on deformable bodies, e.g. ligament insertion and origin sites, to relevant rigid body as boundary conditions to tie the nodes to rigid bodies.
    - Setting of six degrees-of-freedom kinematics (translations and rotations) of all rigid bodies to fixed as a place holder. Note that FEBio assigns these boundary conditions at the center of mass of the rigid body.[13]
- Load Data – define the load curves that were used; "linear", and "steps"
- Output requests – "contact gap", "contact pressure", "contact traction", "displacement", "reaction forces", "stress"
- Module – set to "solid" for solid mechanics analysis

- Step and control parameters – selected parameters to set simulation configuration for implicit static analysis using quasi-Newton incrementation with output at desired intervals using two simulation steps (“Initialize” and “LoadingStep”) as place holders.
    - time_steps = steps (default for steps is 10)
    - step_size = 1./steps
    - dtmin = 1e-10
    - dtmax = 1./steps
    - max_retries = 20
    - opt_iter = 10
    - aggressiveness = 1
    - optimize_bw = 1
    - plot_level = PLOT_MUST_POINTS
    - analysis = static
    - min_residual = 1e-10

Many of the FEBio parameters set above are default values. For parameters that are not specified, FEBio defaults[13] were used. Iterations of these parameters are possible depending on convergence of simulations.

Model Customization

Target outcome Updated model for finite element analysis in FEBio[13] format (.feb[13], XML[20] based text file) with modified tissue-specific constitutive models, additional joint stabilizers and convenience structures, implementation of in situ strain, implementation of anatomically based coordinate system and kinematic chains, modified loading and boundary conditions and, output requests to quantify joint kinematics-kinetics and tissue mechanics relevant to simulation of passive knee flexion ; generated from template model file (.feb)[13].

Software requirements FebCustomization_p3.py**.** In house script to modify template knee joint model to match the specifications of model development. Requires an XML[20] file containing the desired model properties, which includes material properties, and manually chosen anatomical

landmarks. To be used with Python[32], source code available at https://simtk.org/plugins/datashare/?group_id=485# (OKS_model) – DOI pending.

AnatomicalLandmarks_p3.py. In house script to utilize anatomical landmarks (described in an XML file containing the desired model properties) to calculate and add anatomical points and definitions to the model. To be used with Python[32], source code available https://simtk.org/plugins/datashare/?group_id=485# (OKS_model) – DOI pending.

Input Template (or customized) model in FEBio[11] format (.feb)[13] and tissue constitutive models and parameters relevant to knee mechanics (described below) as a text based input file.

1. Constitutive Models and Parameters

Material properties and the constitutive models were adapted from literature as described below.

Bone All bones (femur, tibia, fibula, patella) were assumed to be rigid bodies. Bones have a much higher stiffness than the other knee tissues. Rigid body assumption simplifies computation, therefore decreasing the computational cost and facilitates definition of joint kinematics and/or kinetics as loading and boundary conditions. A density of 1e-9 tonnes/mm^3 (identical to water; consistent with spatial units of mm). It should be noted that density assignment did not have importance on static simulations without the action of gravity. It is provided as a place holder.

Cartilage Cartilage was modeled as a nearly incompressible Neo-Hookean material defined by FEBio[13] setting C2 parameter of FEBio material type Mooney-Rivlin (uncoupled)[12]. This is a fairly simplified representation of cartilage's mechanical behavior. We believe that it would be adequate and computationally less challenging for joint level simulations while providing an opportunity to understand local mechanical environment on and within the cartilage. Cartilage constitutive model and coefficients were similar to previous modeling study[10], which reported an elastic modulus of 15 MPa and Poisson's ratio of 0.475. Corresponding Neo-Hookean material coefficients are noted below.

**Density* (tonnes/mm^3)**	**C1 (MPa)**	**C2 (MPa)**	**K (MPa)**
1e-9	2.54	0	100

All units are consistent with spatial units of mm.

*Density assignment is a place holder; it did not have importance on static simulations without the action of gravity.

Ligaments and Tendons Ligaments and tendons were modeled as nearly incompressible, transversely isotropic, hyperelastic material with a Mooney-Rivlin ground substance (Neo-Hookean by setting C2 = 0). This type of representation accommodates tensile dominant behavior of the ligaments dictated by their fiber alignment across their longitudinal axis. The parameters were identical to a previous modeling study[21], which fitted data from literature. These values are noted below.

Ligament	Density* (tonnes/mm^3)	C1 (MPa)	C2 (MPa)	K# (MPa)	C3 (MPa)	C4	C5 (MPa)	λ_m
ACL	1e-9	1.95	0	146.41	0.0139	116.22	535.039	1.046
PCL	1e-9	3.25	0	243.9	0.1196	87.178	431.063	1.035
MCL	1e-9	1.44	0	793.65	0.57	48.0	467.1	1.063
LCL$	1e-9	1.44	0	793.65	0.57	48.0	467.1	1.063
PL	1e-9	2.75	0	206.61	0.065	115.89	777.56	1.042
QT&	1e-9	2.75	0	206.61	0.065	115.89	777.56	1.042

All units are consistent with spatial units of mm.

ACL: anterior cruciate ligament. PCL: posterior cruciate ligament. LCL: lateral collateral ligament. MCL: medial collateral ligament. PL: patellar ligament. QT: quadriceps tendon.

*Density assignment is a place holder; it did not have importance on static simulations without the action of gravity.

#Bulk modulus (K) is calculated as (K = 1/D); D obtained from source literature.

$LCL properties were assumed to be identical to MCL.

&QT properties were assumed to be identical to PL. This assumption should not have significance as the anticipated use of QT is to transfer loads to patella in a distributed manner.

Constitutive modeling of the ligaments requires specification of a fiber direction. In FEBio[12], under the material definition, fiber type can be specified as "vector" so that the initial direction of all fibers in the material point are along the direction of the vector. For each ligament and

tendon this direction was defined as the direction of the longest edge of the oriented bounding box of the tissue to approximate the longitudinal alignment of the tissue.

Meniscus Menisci were modeled as nearly incompressible, transversely isotropic, hyperelastic material with a Mooney-Rivlin ground substance (Neo-Hookean by setting C2 = 0). This material model is seemingly more complicated than transversely orthothropic linear elastic models in literature. Yet, it allowed a convenient way to represent the behavior of meniscus which is largely dictated by its circumferential stiffness (based on fiber alignment) with the capacity to sustain compressive loading. For convenience, the parameters were identical to those used in a previous modeling study of meniscus[28] and they are noted below.

	**Density*** **(tonnes/mm³)**	**C1 (MPa)**	**C2 (MPa)**	**K# (MPa)**	**C3 (MPa)**	**C4**	**C5 (MPa)**	**λ_m**
**Meniscus**	1e-9	4.61	0	92.16	0.1197	150.0	400.0	1.019

All units were consistent with spatial units of mm.

*Density assignment is a place holder; it did not have importance on static simulations without the action of gravity.

#Bulk modulus (K) is calculated as (K = 1/D); D obtained from source literature. It should be noted that due to differences in dilatational component of constitutive models (used in here and in source literature), equivalence of K is an approximation.

Constitutive modeling of the menisci required specification of fiber direction. In FEBio, fiber direction can be specified for each element individually, in the ElementData section[13]. The meniscus fibers were oriented to the oval fitted to the bounding box.

2. Components for Stabilization

The following components were not tissues of interest, however they were incorporated in the model primarily to stabilize patella and represent the extensor mechanism. Mechanical springs and kinematic joints were used to represent these anatomical structures.

Quadriceps Tendon Attachment The quadriceps tendon was attached to the femur proximally. Since the quadriceps muscles are not being modeled, this attachment were represented by a spring. The use of a spring instead allowed a weakly constrained movement of the proximal quadriceps tendon, while still preventing some separation of patella from the femur during

flexion. The spring adds a small force pulling the patella to remain in contact with the femur bone. A spring constant of 1 N/mm was chosen, but can be calibrated if needed. The spring attached to the imaginary rigid body at the top of the quadriceps tendon on one end, and the femur bone on the other. The insertion origin on both rigid bodies was chosen as the origin of the imaginary rigid body.

Patellofemoral Joint Ligaments The medial and lateral patellofemoral ligaments (MPFL, LPFL) were modeled as discrete elements and using the force-displacement curve were defined as a tension-only linear springs.

. The insertion points of the patellofemoral ligaments were located as follows:

- MPFL femoral insertion: 0.5xCD from the distal side of the medial condyle, and 0.4xCD from the posterior side or the medial condyle, where CD is the anterior-posterior size of the medial condyle[(ref)]
- MPFL patellar insertion: superomedial aspect of patella (~ top 1/3)[2]
- LPFL femoral insertion: 10.6 mm anterior, and 2.6 mm distal to the lateral epicondyle, average width 11.7 mm
- LPFL patellar insertion: 8 mm from superior pole to upper insertion, insertion width ~ 45% of articular surface.

Coordinates of anatomical locations, e.g, femoral epicondyles and patellar regions, were located in the MRI if their identification proved to be difficult to find on the meshes.

3. <u>Application of In Situ Strain</u>

Prestrain formulation of FEBio PreStrain Plugin is described in detail in literature[18]; from this literature:

> "The gradient of the local mapping from the stress-free to the prestressed reference configuration is represented by F_p, which will be referred to as the *prestrain gradient*. The total elastic deformation gradient Fe is determined by the composited deformation gradient, "

To scale the in situ stretch as expected when reducing the step size , a load curve was defined for each prestrain ligament to set in situ stretch to 1 (no strain) at time = 0. For example, if the desired initial stretch value is 1.034, the load curve should be:

<loadcurve id="1" type="linear">

<point>0,1.0</point>

<point>1,1.034</point>

</loadcurve>

To use this load curve appropriately, the initial stretch defined in the material was changed to 1.0, e.g.

<stretch lc="1">1.0</stretch>

One average initial stretch was defined for all fibers in each ligament as:

**Ligament**	**Initial Stretch**
ACL*	1.016
PCL#	1.0
MCL*	1.034
LCL*	1.027
PL#	1.0
QT#	1.0

1.0 indicates strain free initial state

*Initial strain was set to average of values reported in the modeling study of Dhaher et al.[9], who referred to previous knee models[21] and experimental data[14].

#Initial strain set to zero due to lack of data, similar to Dhaher et al.[9]

In the prestrain constraint, min_iters and max_iters were set to 0, essentially eliminating augmentation. Simulations solely rely on penalty based constraints to balance the higher possibility of convergence with potentially decreased constraint enforcement.

Since the prestrain update rule is chosen to eliminate distortion, the above values would only be used as an initial guess for fiber stretch. Due to changes in cross sectional area of the ligament, in order to maintain equilibrium, the solver updates the fiber stretches as needed. Due to this fact, it may be unnecessary for the modeler to define “anterior”, “posterior” etc. regions (as done in previous studies[9,21]), as the values are likely to change. Thus, one average value was given as the initial guess for fiber stretch for all regions in the ligament. After the solver determined the updated initial stretch values, they can be compared to the above literature values.

4. Locating Joint Axes

Tibiofemoral Joint The Grood and Suntay Joint Coordinate System[15] (JCS) was used to define this joint. This method requires a definition of a tibial coordinate system (x_T,y_T,z_T), a femoral coordinate system (x_F,y_F,z_F), and a floating axis (F_{TF}). The following anatomical landmarks were located on the meshes of the femur and tibia:

- medial tibial spine (intercondylar eminence)
- lateral tibial spine (intercondylar eminence)
- approximate center of medial tibial plateau
- approximate center of lateral tibial plateau
- most distal point on the posterior surface of the femur, midway between the medial and lateral condyles
- medial femoral condyle (most distal point on posterior surface)
- lateral femoral condyle (most distal point on posterior surface)

Tibial Coordinate System:

Tibial Origin: Mid-point between tibial spines (medial and lateral intercondylar eminences).

Tibial Mechanical Axis (z_T-axis): JCS[15] defines this axis as passing through the midpoint between the tibial spines proximally, and the center of the ankle distally. Due to a lack of MRI of the ankle, we assumed this axis to pass through the same point proximally (midpoint between spines), and extend parallel to the z- direction of the MRI coordinate system (approximately aligned with the longitudinal axis of the body). The z-axis is positive in the proximal direction.

Tibial Anterior Axis (y_T-axis): The cross product between the z_T-axis, and a line connecting the approximate center of each tibial plateau. The y-axis is positive in the anterior direction.

Tibial Mediolateral Axis (x_T-axis): The cross product between the y_T-axis and the z_T-axis.

Femoral Coordinate System:

Femoral Origin: Most distal point on the distal femur, midway between the medial and lateral condyles.

Femoral Mechanical Axis (z_F-axis): JCS[15] defines this axis as passing through the center of the femoral head proximally, and the most distal point on the posterior surface of the femur distally. Due to lack of MRI data of the femoral head, we assumed the axis to pass through the same point distally (distal point on posterior surface), and extend parallel to the z-direction of the MRI coordinate system (approximately aligned with the longitudinal axis of the body). The z_F-axis is positive in the proximal direction.

Femoral Anterior Axis (y_F-axis): The cross product between the femoral mechanical axis (z_F-axis) and a line connecting the femoral condyles. The y_F-axis is positive in the anterior direction.

Flexion Axis (x_F-axis): The cross product between the y_F-axis and the z_F-axis.

Tibiofemoral Floating Axis:

The tibiofemoral floating axis (F_{TF}-Axis) is defined as the cross product between the z_T-axis and the x_F-axis at any given joint position.

Patellofemoral Joint This joint motion is described in a method similar to JCS[15], according to Bull et al.[5] This method requires a definition of a patella coordinate system (x_P,y_P,z_P) , femoral coordinate system (same as that described above for the tibiofemoral joint), and a Floating axis (F_{PF}). The following anatomical landmarks were located on the mesh of the patella:

- medial patella ridge
- lateral patella ridge
- midpoint of patella in coronal view

Patella Coordinate System:

Patella Origin: Mid-point of medial and lateral ridges of patella.

Medial-Lateral Axis (x_P-axis): The line connecting the medial and lateral ridges of the patella.

Superior-Inferior Axis (z_P-axis): Perpendicular to x_P and through the midpoint of the patella in coronal view(ref).

Anterior-Posterior Axis (y_P-axis): The cross product between x_P-axis and z_P-axis.

Patellofemoral Floating axis:

The patellofemoral floating axis (F_{PF}-axis) is defined as the cross product between the z_P-axis and the x_F-axis.

Note:. We defined the superior-inferior axis as perpendicular to zp, and in line with the coronal image plane (y=0). This would not work if the knee is rotated in the MRI coordinate system. If this is the case, an additional anatomical landmark at the "top" of the patella can be chosen to help align the axis, instead of "midpoint of patella in coronal view". As long as the patello-femoral joint constraints are all set free, this should not affect the model, as the joint has 6 degrees of freedom.

Creating Kinematic Joints in FEBio Recent versions of FEBio[13] do not allow independent prescription of rotational degrees of freedom for a rigid body. For this reason and to facilitate loading and boundary conditions relevant to anatomy of the knee, kinematic joint chains were defined for tibiofemoral and patellofemoral joints.

Tibiofemoral Joint:

Three rigid cylindrical joints[13] were added to the constraints section to define tibiofemoral motion. For this reason two "imaginary" rigid bodies were defined:

- TFTO: located at the origin of the tibial (x_T,y_T,z_T) coordinate system.
- TFFO: located at the origin of the femoral (x_F,y_F,z_F) coordinate system.

The cylindrical joints were defined as follows:

**Motion**	**joint_origin**	**joint_axis**	**body_a**	**body_b**
**Flexion-extension**	Origin of (x_F,y_F,z_F)	x_F-axis	Femur	TFFO
**External-internal rotation**	Origin of (x_T,y_T,z_T)	z_T-axis	TFTO	Tibia

**Abduction-adduction**	Intersection of F_{TF}- and x_F-axes	F_{TF}-axis	TFFO	TFTO

All other parameters in the rigid cylindrical joints were set to FEBio defaults[13]. Each cylindrical joint also had a translational component to describe tibiofemoral joint translations[13].

Patellofemoral Joint:

Three rigid cylindrical joints[13] were added to the constraints section to define patellofemoral motion. For this reason, two more "imaginary" rigid bodies were defined:

- PFPO: located at the origin of the patella (x_P,y_P,z_P) coordinate system.
- PFFO: located at the origin of the femoral (x_F,y_F,z_F) coordinate system.

The cylindrical joints were defined as follows:

**Motion**	**joint_origin**	**joint_axis**	**body_a**	**body_b**
**Patellar flexion and shift**	Origin of (x_F,y_F,z_F)	x_F-axis	Femur	PFFO
**Patellar tilt**	Origin of (x_P,y_P,z_P)	z_P-axis	PFPO	Patella
**Patellar rotation**	Intersection of F_{PF}- and x_F-axes	F_{PF}-axis	PFFO	PFPO

All other parameters in the rigid cylindrical joints were set to FEBio defaults. Each cylindrical joint also had a translational component to describe patellofemoral joint translations[13].

Note: Centers of mass of tibia, patella, femur rigid bodies were moved from world origin to their respective coordinate system origins, in order to allow for easier manipulation while debugging the model, and for easier calculations in post-processing.

5. <u>Loading and boundary conditions</u>

The simulation strategy was to use a one step solution. Load curves defined the in situ strain (prestrain) application from time 0 to 1, then hold the prestrain at its final value between time 1 and 2. The loading (flexion) occurs from time 1 to 2, and the load curve for the flexion is set to be zero from time 0 to 1, i.e., fixing the flexion angle during the prestrain step.

In Situ Strain Application In situ strains were applied based on previously described specifications (see above). Any loading and boundary conditions that are not specified below were set to FEBio defaults[13].

- Prestrain: as described in in situ strain application section (see above)
- Femur: All degrees of freedom (3 translations, 3 rotations) free.
- Tibia: All degrees of freedom (3 translations, 3 rotations) fixed.
- Fibula: All degrees of freedom (3 translations, 3 rotations) fixed.
- Patella: All degrees of freedom (3 translations, 3 rotations) free.
- Tibiofemoral cylindrical joints: Flexion fixed (at 0º); remaining degrees of freedom (3 translations, 2 rotations) free.
- Patellofemoral cylindrical joints: All degrees of freedom (3 translations, 3 rotations) free.
- Quadriceps tendon slider joint: All degrees of freedom (3 translations, 3 rotations) free.

Passive Flexion At the start of the passive flexion application, in situ strains should have already been applied as prescribed. Our interpretation of passive knee flexion is that the motion of the knee is guided by joint contact and connective tissue recruitment. Therefore no external loading was applied; the movement of the knee was unconstrained other than prescription of the flexion angle up to 90º. Any loading and boundary conditions that are not specified below were set to FEBio defaults[13].

- Femur: All degrees of freedom (3 translations, 3 rotations) free.
- Tibia: All degrees of freedom (3 translations, 3 rotations) fixed.
- Fibula: All degrees of freedom (3 translations, 3 rotations) fixed.
- Patella: All degrees of freedom (3 translations, 3 rotations) free.
- Tibiofemoral cylindrical joints: Flexion prescribed (0º to 90º during simulation step); remaining degrees of freedom (3 translations, 2 rotations) free.
- Patellofemoral cylindrical joints: All degrees of freedom (3 translations, 3 rotations) free.
- Quadriceps tendon slider joint: All degrees of freedom (1 translation) free.

6. <u>Simulation output requests</u>

By default, FEBio[13] provides two output files: .xplt, plotfile, a binary file that includes all default and requested field variables compatible with PostView[22]; .log, logfile, a text file that reports the

convergence history of the simulation along with convergence metrics including any other variables requested for output. In accordance with the overall customization of the model and to provide output metrics relevant to simulation case, output of the following metrics were requested:

- Fiber stretch – fiber stretch during simulation in tissues with compatible constitutive models (ligaments, tendon, menisci).
- Generalizable parameters – contact gap, pressure, traction; displacement; reaction forces; stress; to be stored in plotfile.
- Prestrain stretch – actually the total fiber stretch, including the effects of the prestrain and deformation; to be stored in plotfile.
- Fiber stretch – fiber stretch due to deformation during simulation in tissues with compatible constitutive models; this differentiation is important to understand ligament deformations when in situ strain is implemented.
- Rigid body data – Request outputs kinematics and kinetics of all rigid bodies including imaginary rigid bodies used for joint coordinate systems; to be stored in plotfile and logfile. Information in logfile can be used to reconstruct joint kinematics.
- Rigid joints data – Request outputs only kinetics but not kinematics as it is not implemented in FEBio[11]; to be stored in plotfile and logfile.

See FEBio User's Manual[13] for more details.

Simulation

Target outcome Solution of fully customized model through finite element analysis using FEBio[11]; generating simulation results as binary and text output files (.xplt and .log, respectively).

Software requirements: FEBio**.** FEBio[11] is a nonlinear implicit finite element analysis framework designed specifically for analysis in biomechanics and biophysics (custom open source license; free for academic research use, licensing for commercial use is available, see http://www.febio.org). The latest version of FEBio[11] (version 2.9 at the time of preparation of this document) was used.

FEBio PreStrain Plugin. PreStrain[18] Plugin provides a general framework for representing prestrain in a finite element model using a prestrain gradient method. A version compatible with

the latest version FEBio was used (at the time of preparation of this document, version 1.0 supporting FEBio 2.9 has been available).

Input Fully customized model in FEBio format (.feb[13]).

Simulation Process Invoked FEBio[13] with the model file as input. For simulations that did not convergence, relaxation of convergence tolerances and utilization of alternative solution algorithms, contact formulations, etc. were employed (see Appendix A6).

Post-Processing

Target outcome Extraction and summary of knee kinematics and kinetics during passive flexion; processed using raw simulation results of fully customized model with FEBio[13] (.log file), supported by graphs as binary image files.

Software requirements LogPostProcessing.py. In house script to read model specific FEBio[13] log file, extract and store joint kinematics-kinetics, and plot joint kinematics-kinetics. To be used with Python, source code available at https://simtk.org/plugins/datashare/?group_id=485# (OKS_model) – DOI pending.

Input Solution of fully customized model through finite element analysis using FEBio[13]; simulation results as binary and text output files (.xplt and .log, respectively).

LogPostProcessing.py reads the log file and extract, store (as .xml[16]) and plot knee kinematics and kinetics during passive flexion (as .png[23]):

- Kinematics of tibiofemoral cylindrical joints: 3 rotations, 3 translations total
- Kinematics of patellofemoral cylindrical joints: 3 rotations, 3 translations total
- Translation of tibia origin relative to femur origin in femoral coordinate system
- Translation of patella origin relative to femur origin in femoral coordinate system
- Constraint moment to maintain the knee joint at prescribed flexion

Visualization PostView[22] was used to take snapshots of the model at different flexion angles, as obtained through simulation of passive flexion. PostView[22] can also be used to inspect tissue stress-strain distributions, export data, images, and animations.

A2. Experimental data processing

Registration for Specimen-Specific Calibration

Target outcome Coordinate system transformation matrices between joint testing and imaging coordinate systems of bones and experimental anatomical landmarks transformed to model coordinate system in XML[20] based text files. Full knee model with joint coordinate system defined to align with the experimental coordinate system, in FEBio[13] format (.feb, XML[20] based text file).

Software requirements Register_probed_points.py – in house script to transform the digitized anatomical landmarks to the image coordinate system. To be used with Python[32], source code available at https://simtk.org/plugins/datashare/?group_id=485# (OKS_model) – DOI pending.

Input Experimental probed points on registration markers and anatomical landmarks and coordinate system transformations (State.cfg); raw registration marker geometries from imaging; FEBio[13] model file.

Registration In the State.cfg file, one can find the coordinates of the probed points on registration markers (three on femur, three on tibia) and bone landmarks collected during mechanical testing[6]. A sphere was fit to probed points on each registration marker to obtain its center in the local bone motion tracking system coordinate system. Similarly, a sphere was fit to raw registration marker geometries obtained by segmentation of imaging data to obtain their centers in image coordinate system. For each cluster of registration markers on the bone, the transformation matrix was calculated between the local bone motion tracking system and image coordinate system using singular value decomposition between sphere centers. In following, anatomical landmarks on each bone, which are probed during mechanical testing, were transformed to image coordinate system to serve as the foundation to redefine model joint coordinate systems and cylindrical joint axes. The coordinate systems of tibia and femur were updated based on descriptions provided in the experiment documentation[16](also see below). Patella coordinate system remained the same due to incomplete data on patella registration marker assembly.

Tibia

T_1= Most lateral point on the tibial plateau

T_2= Most medial point on the tibial plateau

T_3= Distal tibia point (medial malleolus of the tibia: most medial point)

T_4= Distal tibia point (medial malleolus of the tibia: most medial point)

T_5= Distal tibia point (lateral malleolus of the tibia: most lateral point)

T_6= Distal tibia point (lateral malleolus of the tibia: most lateral point)

tibial origin:

Tibia z-axis : , where

Tibia y-axis: , normalized; where

Tibia x-axis: , normalized

Femur

F_1= Lateral femoral epicondyle

F_2= Medial femoral epicondyle

F_3= Proximal femur point

F_4= Proximal femur point

F_5= Proximal femur point

F_6= Proximal femur point

Femur origin:

Femur x_axis:

Femur y_axis:

Femur z_axis:

Customized Full Knee Model with Experiment Coordinate Systems Experimental landmarks were used to define anatomical joint coordinate system in the model. The tibiofemoral floating

axis (FTF-axis) was defined as the cross product between the T_z-axis and the F_x-axis at any given joint position. The patellofemoral floating axis (FPF-axis) was defined as the cross product between the P_z-axis and the F_x-axis. The in house script FebCustomization_p3.py needs to be run for this purpose. The model were therefore aligned with the experiment such that the axes as defined in the experiment are the same as the ones defined in the model (For script usage, see Appendix A8).

Specimen-Specific Kinematics-Kinetics Data Processing

Target outcome Experimental kinematics-kinetics data transformed, reduced, and presented in a form amenable for simulations with the full knee model, as text files (.csv)[27] and graphs as binary images (.png)[23]. Representation of experimental kinematics-kinetics were separated for passive flexion and for laxity data (as a function of target flexion angle, dominant degree of freedom, loading direction in the dominant degree of freedom).

Software requirements tdms_processing_oks.py – in house script to process the tdms[31] files provided with Open Knee(s)(ref) experimental data. To be used with Python[32], source code available at https://simtk.org/plugins/datashare/?group_id=485# (OKS_model) – DOI pending.

Input Experimental joint kinematics-kinetics data (.tdms), joint coordinate system offsets (State.cfg); coordinate system transformation matrices between joint testing and imaging, and experimental anatomical landmarks transformed to model coordinate system (.xml).

Processing of Passive Flexion Data Experimental passive flexion data file (.tdms) were processed. Kinematics data were extracted from "State.Knee JCS" channels in the data files[3], which provide joint kinematics in an anatomical joint coordinate system (defined in the experiment based on cylindrical joints)[16] relative to a reference state (joint offsets given in State.cfg[4]). Kinetics data were extracted from "State.JCS Load" channels in the data files[3], which provide joint kinetics in an anatomical tibia coordinate system as applied as loads on tibia[16]. The Python script extracted, processed, and stored the data:

1. Extracted data such that the data from 0° to maximum flexion was re-sampled at 5° increments, averaging data on each channel where flexion angle was within +/- 0.5 °.
2. Added kinematics offsets (from State.cfg) to kinematics channels to report bone pose and orientation in an absolute fashion.
3. Transformed kinematics data to the convention used in the model, i.e., cylindrical joint translations and rotations, accommodating offsets at model reference state when reconstructing experiment coordinate systems in the model.

4. Transformed kinetics data to the convention used in the model, i.e., joint loading applied to femur in model coordinate system, which is registered and aligned to experiment coordinate system.
5. Wrote kinematics and kinetics to a text based file (.csv) both in experiment and model conventions; plot and store as graphics files (.png).

Thresholds for cropping and resampling of data may change depending on data quality, e.g., noise and errors that may become apparent during analysis. The content of experimental kinematics-kinetics data files are in right knee abstraction, which were managed during any coordinate system transformation.

Processing of Laxity Data Kinematics-kinetics data for laxity were split into files based on flexion angle (0º, 30º, 60º, 90º), dominant loading axis (anterior-posterior translation, internal-external rotation, varus-valgus), and direction of loading axis (positive, negative). Kinematics data were extracted from "State.Knee JCS" channels in the data files[3], which provide joint kinematics in an anatomical joint coordinate system (defined in the experiment based on cylindrical joints)[16] relative to a reference state (joint offsets given in State.cfg[4]). Kinetics data were extracted from "State.JCS Load" channels in the data files[3], which provide joint kinetics in an anatomical tibia coordinate system as applied as loads on tibia[16]. A Python script was developed for extraction, processing, and storage of laxity data:

- Extracted data by finding the indices of the data points (from Kinetics.JCS.Desired) where the force was held constant for all loading cases at all degrees of flexion where laxity data was collected (0 º, 30 º, 60 º, 90 º), taking the index of the final data point for each flat section of the data and extracting the actual kinetics and kinematics data from 'State.JCS Load' and 'State.Knee JCS' using the indices.
- Added kinematics offsets (from State.cfg) to kinematics channels to report bone pose and orientation in an absolute fashion.
- Transformed kinematics data to the convention used in the model, i.e., cylindrical joint translations and rotations, accommodating offsets at model reference state when reconstructing experiment coordinate systems in the model.
- Transformed kinetics data to the convention used in the model, i.e., joint loading applied to femur in model coordinate system, which is registered and aligned to experiment coordinate system.
- Wrote kinematics and kinetics to a text based file (.csv) both in experiment and model conventions; plot and store as graphics files (.png).

Thresholds for cropping and resampling of data may change depending on data quality, e.g., noise and errors that may become apparent during analysis. The content of experimental kinematics-kinetics data files are in right knee abstraction, which were managed during any coordinate system transformation.

A3. Model customization for application of kinematics-kinetics (with or without experimental data)

Target Outcome Customized full knee models in FEBio[13] format (.feb, XML[20] based text file) prepared for all simulation cases, and all experimental loading conditions. Models include converged meshes, confirmed material properties and calibrated in situ ligament strains, and for reproduction of experiments, loading and boundary conditions of joint testing registered and transformed to model coordinate system.

Software requirements experiment_to_model.py: In house Python script to update models with target kinetics-kinematics. Script available in, https://simtk.org/plugins/datashare/?group_id=485# (OKS_model) – DOI pending.

Input Template FEBio model file of the full knee (.feb[13]) and model properties (.xml[20]) files for with converged meshes, confirmed material properties, experiment coordinate systems, and calibrated in situ ligament strains; kinematics-kinetics data (experimental or simulated target) (.csv[27]).

Customization for Experiment or simulated Loading Cases Python script updates knee model in FEBio[13] to replicate experimental or simulated conditions. Experimental kinetics were applied as external femur loads, and experiment flexion angle was prescribed to the extension-flexion joint. Tibia was fixed; femur and patella were free to move and all loads and boundary conditions were applied in one step. From time 0 to 1, in situ strain were applied while keeping flexion at 0°. From time 1 to 2, the loads and boundary conditions at the start of experiment were prescribed, i.e., the flexion angle was set and the loads in the remaining degrees of freedom were applied on femur. From time 2 to 3, the loads and boundary conditions of the experimental trial were applied until the end point of the experiment. Load curves for each degree of freedom (particularly the dominant loading) were defined based on experiment data points and simulation output were requested at each experiment point. The kinematics-kinetics trajectories of experiment were split to facilitate prescription of loading scenarios in simulations.

A4. Model calibration procedure

Mesh Convergence

Target Outcome Geometric reconstruction of tissues of interest as smooth and watertight triangulated surface representations (.stl)[29] and finite element meshes (.med)[24] with several mesh densities including converged mesh densities. Compartmental models of tissues of interest in FEBio[13] format (.feb, XML[20] based text file) for mesh convergence simulations. Simulation results as binary and text output files (.xplt and .log, respectively)[13] and as summary of mesh convergence analysis including target convergence metric as a function of mesh density (XML[20] based text file). Full knee model with converged meshes in FEBio[13] format (.feb, XML[20] based text file). Tissues for which mesh convergence were conducted include cartilage – femoral, tibial (medial & lateral), patellar; menisci – medial & lateral, ligaments – anterior/posterior cruciate, medial/lateral collateral, patellar; tendons – quadriceps.

Input Raw triangulated surface representations of tissues of interest (without filtering and smoothing) in .stl[29] format in image coordinate system.

Geometry Generation Procedures For each tissue of interest, several geometries were created at different mesh densities. Smoothing procedures were performed as described in model development A1. The Iso Parameterization Remeshing phase was repeated several times, to obtain the mesh densities specified below.

	**Iso Parameterization Sampling Rates**
**Femoral Cartilage**	8,10,15,20
**Tibial Cartilage**	6,8,10,15
**Patellar Cartilage**	6,8,10,15
**Menisci**	4,6,8,10
**ACL**	4,6,8,10
**PCL**	3,4,6,8
**Patellar Ligament**	4,6,8,10
**Quadriceps Tendon**	4,6,8,10

	Iso Parameterization Sampling Rates
**MCL**	5,7,9,11
**LCL**	3,4,6,8

ACL: anterior cruciate ligament. PCL: posterior cruciate ligament. LCL: lateral collateral ligament. MCL: medial collateral ligament.

Mesh Generation Procedures For each tissue geometry that was generated above, a mesh was created in Salome[26]. A new MED[24] file was generated where all node/element/face groups, which were already created for original tissue mesh, were transferred to the new MED[24] file. This was done in a scripted fashion, using in house Python[32] script transfer_med_groups.py by treating the groups as a binary field and applying that binary field to the new mesh to find the correspondence of nodes and elements in the new mesh. This correspondence was used to select nodes and elements to generate groups with the new node and element sets. A quality assurance check was completed here to ensure that all node/element/face groups are as expected. For example, a check was done for all ligament insertion origins that the insertion area covers the entire width of the ligament where it connects to the bone.

Model Generation Procedures Each of the tissues of interest was tested by creating a model with the boundary conditions outlined in the table below. The models were created for each mesh density, such that 4 models exist for each tissue, with the only difference being the density of the mesh of the tissue of interest. Model generation was performed according to the model development A1 (using in home scripts MedToFebio.py, FebCustomization_p3.py), and boundary conditions were updated manually in the FEBio model input files for each model.

Tissue of Interest	Included Parts	Boundary Conditions*	Simulation Outputs
**Femoral Cartilage**	FMC, FMB, TBC-L, TBC-M	Compression of the FMC between the FMB (rigid) and the TBC-L, TBC-M modeled as rigid bodies. TBC fixed, FMB displaced 2 mm in -z direction.	Z-reaction force in FMB (primary)<br><br>Contact pressure (secondary)
**Tibial Cartilage**	1. FMB, TBC-L, TBB<br><br>2. FMB, TBC-M, TBB	Compression of the Tibial Cartilage between the TBB (rigid) and the FMC modeled as a rigid body. TBB fixed,	Z-reaction force in FMC (primary)

**Tissue of Interest**	**Included Parts**	**Boundary Conditions***	**Simulation Outputs**
		FMC displaced 1 mm in the -z direction for TBC-L and 0.2 mm for TBC-M.	Contact pressure (secondary)
**Patellar Cartilage**	PTC, PTB, FMC	Compression of the PTC between the PTB (rigid) and the FMC modeled as a rigid body. FMC fixed, PTB displaced 1mm in the y direction.	Y-reaction force in PTB (primary)<br><br>Contact pressure (secondary)
**Menisci**	1. MNS-M, FMC, TBC-M, TBB<br><br>2. MNS-L, FMC, TBC-L, TBB	Compression of the MNS between the FMC and TBC, both modeled as rigid bodies.<br><br>TBC fixed, FMC displaced 1 mm in the -z direction. Test applied to MNS-M, MNS-L separately.	Z-reaction force in FMC (primary)<br><br>Contact pressure (secondary)<br><br>Fiber stretch (secondary)
**ACL**	ACL, FMB, TBB	Tension test. TBB fixed, FMB displaced 3 mm in the z direction.	FMB reaction force (primary)<br><br>Fiber stretch (secondary)
**PCL**	PCL, FMB, TBB	Tension test. TBB fixed, FMB displaced 3 mm in the z direction.	FMB reaction force (primary)<br><br>Fiber stretch (secondary)
**Patellar Ligament**	P TL, PTB, TBB	Tension test. TBB fixed, PTB displaced 4 mm in the z direction.	Z-reaction force in PTB (primary)<br><br>Fiber stretch (secondary)

Tissue of Interest	Included Parts	Boundary Conditions*	Simulation Outputs
**Quadriceps Tendon**	QAT, PTB, QSO (rigid body connected to QAT)	Tension test. QSO fixed, PTB displaced 4 mm in the z direction.	Z-reaction force in PTB (primary)<br><br>Fiber stretch (secondary)
**MCL**	MCL, TBB, FMB	Tension test. TBB fixed, FMB displaced 4 mm in the z direction	Z-reaction force in FMB (primary)<br><br>Fiber stretch (secondary)
**LCL**	LCL, TBB, FBB	Tension test. FBB fixed, FMB displaced 4 mm in the z direction	Z-reaction force in FMB (primary)<br><br>Fiber stretch (secondary)

FMB: femur bone. TBB: tibia bone. FBB: fibula bone. PTB: patella bone. FMC: femoral cartilage. TBC-L: tibial lateral cartilage. TBC-M: tibial medial cartilage. PTC: patellar cartilage. MNS-L: lateral meniscus. MNS-M: medical meniscus. ACL: anterior cruciate ligament. PCL: posterior cruciate ligament. MCL: medial collateral ligament. LCL: lateral collateral ligament. PTL: patellar ligament. QAT: quadriceps tendon. QSO: quadriceps origin.

*Displacement levels were chosen to induce approximately 10% nominal strain on target tissue.

Mesh Convergence Procedures Each of the models created above was run in FEBio, beginning with the coarsest mesh density. The simulation was repeated, each time with a finer mesh. The primary measured outputs were compared with those of the previous simulation, and when there was less than 5% difference (7.6% for medial tibial cartilage), convergence was assumed. The mesh density at which the output converged was then used in the final model. If no convergence is found within the specified mesh densities, finer/coarser mesh densities were created to continue testing until convergence was found.

Template Full Knee Model with Converged Meshes The in house scripts MedToFebio.py, and FebCustomization_p3.py were run including all of the selected knee tissues with converged mesh densities.

Confirmation of Material Properties

Target Outcome Compartmental models of tissues of interest in FEBio[13] format (.feb, XML[20] based text file) for simulations to confirm and modify material properties using converged meshes. Simulation results as binary and text output files (.xplt and .log, respectively)[13] and as summary of calibration process including target metric, model predictions as a function of material property and fit error (XML[20] based text file). Full knee model with confirmed and modified material properties in FEBio[13] format (.feb, XML[20] based text file). Tissues for which material properties were confirmed include cartilage – femoral, tibial (medial & lateral), patellar; menisci – medial & lateral, ligaments – anterior/posterior cruciate, medial/lateral collateral, patellar; tendons – quadriceps.

Input FEBio model file of the full knee with converged meshes, including all the tie/contact surfaces.

Compartmental Modeling of Structural Tissue Behavior In order to confirm and modify material properties, structural tissue response or gross material behavior were compared with literature. If, the tissue behavior fell outside the range of expected behavior, the material properties were adjusted until the mechanical response of the tissue is considered within the reported normal range. This process essentially confirmed each tissue material property used for model development with a second information resource. Details of the simulations (and/or analyses) performed are provided below. An in house script ModelReduction_rigid3.py, was used to reduce the full knee model to include only the desired components for each of the test simulation cases. An in house script StiffnessFromLog.py was used to determine linear stiffness from model results.

Ligaments and Tendons

**Tissue to Calibrate**	**Included Parts**	**Boundary Conditions***	**Simulation Outputs**	**Expected Behavior**
**ACL**	ACL, FMB, TBB	Tension: TBB fixed, FMB displaced 3 mm in the fiber direction of the ACL.	FMB reaction force-displacement curve	Linear stiffness = 242±28 N/mm[14]

Tissue to Calibrate	Included Parts	Boundary Conditions*	Simulation Outputs	Expected Behavior
**PCL**	PCL, FMB, TBB	Tension: TBB fixed, FMB displaced 5 mm in the fiber direction of the PCL.	FMB reaction force-displacement curve	Linear stiffness = 258±62 N/mm[15]
**Patellar Ligament**	PTL, PTB, TBB	Tension: TBB fixed, PTB displaced 5 mm in the fiber direction of the PTL.	PTB reaction force-displacement curve	Linear modulus = 337.8.±67.7 MPa[17]
**Quadriceps Tendon**	QAT, PTB, QSO	Tension: QSO fixed, PTB displaced 5 mm in the fiber direction of the QAT.	PTB reaction force-displacement curve	Linear modulus = 255.3±64.1 MPa[17]
**MCL**	TBB, MCL, FMB	Tension: TBB fixed, FMB displaced 5 mm in the fiber direction of the MCL.	FMB reaction force-displacement curve	Linear stiffness = 63±14 N/mm[16]
**LCL**	FBB, FMB, LCL	Tension: FBB fixed, FMB displaced 5 mm in the fiber direction of the LCL	FMB reaction force-displacement curve	Linear stiffness = 59±12 N/mm[16]

FMB: femur bone. TBB: tibia bone. FBB: fibula bone. PTB: patella bone. ACL: anterior cruciate ligament. PCL: posterior cruciate ligament. MCL: medial collateral ligament. LCL: lateral collateral ligament. PTL: patellar ligament. QAT: quadriceps tendon. QSO: quadriceps origin.

*Displacement levels were chosen to induce approximately 10% nominal strain on target tissue. No prestrain was applied.

For the tension test simulations in the fiber direction, a load curve was defined from 0 to X, where X is the desired total displacement. Then, in the boundary section the prescribed displacement

was specified in the fiber direction. For example, given a fiber direction of [-0.101,-0.511,0.854] a unit displacement (X=1) :

```
<rigid_body mat="4">

<prescribed bc="x" lc="3">-0.101</prescribed>

<prescribed bc="y" lc="3">-0.511</prescribed>

<prescribed bc="z" lc="3">0.854</prescribed>

<fixed bc="Rx"/>

<fixed bc="Ry"/>

<fixed bc="Rz"/>

</rigid_body>
```

Python script StiffnessFromLog.py was used to extract rigid body reaction force – displacement data from simulation output (.log). In following, force-displacement response of the tissue along its fiber direction was calculated. Linear stiffness of the tissue (k) was calculated by fitting a line to the linear portion (upper third) of the tissue's simulated structural response. Linear modulus (E) was calculated as (k x L_o / A_o), where L_o and A_o are the reference ligament length and cross sectional area, respectively. The reference length was calculated as the distance between the centers of the insertion and origin. The cross sectional area was calculated as the average cross sectional area between the insertion and origin. If the simulated tissue properties (k and/or E) fall outside two standard deviations of the expected behavior, the fiber modulus (C5) was scaled to bring the tissue response within the expected range.

<u>Cartilage</u>

Since we assume cartilage properties to be consistent for all cartilage tissues, we performed one indentation test to confirm if cartilage structural response is within what is reported in literature. For this purpose, we used indentation stiffness of lateral tibial cartilage, which was reported as 20.38±5.32 N/mm[30]. A model was generated to reproduce the experiment conditions[30]. The model included the tibia bone (TBB), lateral tibial cartilage (TBC-L), and a 1mm diameter indenter as a rigid body, which was in frictionless contact with the cartilage. The indenter was placed above the cartilage near the meniscus, as described in the study, and a load of 0.5 N was applied

to force the indenter against the tibial cartilage. StiffnessFromLog.py was used to extract indenter force and displacement data from simulation results (.log) and the linear stiffness was calculated using the linear region (upper third) of the force-displacement curve. Material properties of cartilage (C1) were scaled as needed to get the range within two standard deviations of the reported stiffness value. Bulk modulus parameter (K) scaled accordingly.

Meniscus

Depending on the location of the sample (anterior, central, posterior) and the thickness of it, circumferential tensile modulus of medial meniscus was reported as 43.4±26.8 MPa to 141.2±56.7 MPa[17]. The fiber modulus (C5) of meniscus was scaled to match within two standard deviations of the reported modulus.

Customized Full Knee Model with Confirmed Material Properties

The in house script FebCustomization_p3.py needs to be run including all of the selected knee tissues with updated material properties.

Calibration of In Situ Ligament Strains

Target Outcome Full knee models with converged meshes, confirmed material properties, joint coordinate system defined to align with the experimental coordinate system, and loading and boundary conditions of experiments selected for calibration in FEBio[13] format (.feb, XML[20] based text file). Simulation results as binary and text output files (.xplt and .log, respectively) and as summary of calibration process including target metric, model predictions as a function of in situ ligament strains and fit error (XML based text file). Full knee model with calibrated in situ ligament strains in FEBio format (.feb, XML based text file). Tissues for which in situ ligament strains were calibrated include ligaments – anterior/posterior cruciate, medial/lateral collateral.

Input Template FEBio model file of the full knee (.feb [1]) and model properties (.xml[20]) files with converged meshes, confirmed material properties, and experiment coordinate systems; processed specimen-specific kinematics-kinetics data (.csv[27]).

Models experiment_to_model.py, was used to generate models representative of the loading and boundary conditions of selected laxity tests to calibrate in situ strains. Only joint laxity data at 0° flexion was used to modify in situ strains for anterior cruciate ligament (ACL), posterior cruciate ligament (PCL), medial collateral ligament (MCL), and lateral collateral ligament (LCL). The decision to use only these data was made to save on computational cost and time. All other loading scenarios include flexion of the joint prior to performing laxity testing, which can be

costly, and often, convergence issues may arise. This way, the calibration could be performed quickly, and the models were unlikely to have any convergence issues.

Template model (.feb) and model properties (.xml) reflective of converged meshes, confirmed material properties, and experiment coordinate systems were the basis for customization of models for calibration. Modifications to the customization script and/or additional scripts were necessary to implement the loading scenarios Overall, application of loading and boundary conditions and output requests was similar to those described in A1 with exceptions noted in here. Tibia was fixed; femur and patella were free to move and all loads and boundary conditions were applied in one step. From time 0 to 1, in situ strain was applied while keeping flexion at 0°. From time 1 to 2, the loads and boundary conditions at the start of experiment were prescribed, i.e., the flexion angle was set and the loads in the remaining degrees of freedom were applied on femur to reflect the loading state of the joint at the start of testing. This step accounted for any offsets in bone configuration between imaging and the experiment and it should be done after the prestrain step as we do not want the in situ strain calibration to be dependent on the orientation of the knee in different experiment trials. From time 2 to 3, the loads and boundary conditions of the experiment were prescribed, i.e., the flexion angle was constant and the loads in the remaining degrees of freedom were applied on femur. Load curves for each degree of freedom (particularly the dominant loading) were defined based on experiment data points and simulation output were requested at each experiment point. A total of 4 models were generated:

**Model Name**	**Flexion (°)**	**Loading from Experiment**	**To Calibrate**
F00_AT_C	0	Anterior laxity	ACL
F00_PT_C	0	Posterior laxity	PCL
F00_VL_C	0	Valgus laxity	MCL
F00_VR_C	0	Varus laxity	LCL

ACL: anterior cruciate ligament. PCL: posterior cruciate ligament. MCL: medial collateral ligament. LCL: lateral collateral ligament.

Calibration Procedure An iterative procedure was used to identify optimal in situ ligament strains in anterior cruciate ligament (ACL), posterior cruciate ligament (PCL), medial collateral ligament (MCL), and lateral collateral ligament (LCL):

1. Use F00_AT_C (with previously modified ACL, PCL, MCL, LCL in situ strains, if any) to find ACL in situ strain by minimizing the difference between model predicted and experimental anterior translation and force.
2. Use F00_PT_C (with previously modified ACL, PCL, MCL, LCL in situ strains, if any) to find PCL in situ strain by minimizing the difference between model predicted and experimental posterior translation and force.
3. Use F00_VL_C (with previously modified ACL, PCL, MCL, LCL in situ strains, if any) to find MCL in situ strain by minimizing the difference between model predicted and experimental valgus rotation and moment.
4. Use F00_VR_C (with previously modified ACL, PCL, MCL, LCL in situ strains, if any) to find LCL in situ strain by minimizing the difference between model predicted and experimental varus rotation and moment.
5. Repeat steps 1-4 until convergence of in situ strains, i.e., stop when absolute change in calculated in situ strain is less than 0.001.

The Python script InSituOptimization.py was developed to update the in situ strain of the target ligament in the model, to read the simulation results (displacement and load in dominant degree of freedom), to implement a scalar (one-dimensional) optimization that minimized the sum of the squared differences between model predicted and experimental loading response in the dominant degree of freedom, and to write optimization results in a text file (.xml). It should be noted that these calculations were performed with readily aligned kinematics-kinetics conventions of the model and experiment, i.e., following registration, kinematics-kinetics data processing, and accounting for experimental local coordinate systems offsets.

A5. Edge lengths calculated for all meshes used in the models

For each of the mesh component in each model, average edge length (mm), standard deviation(mm), number of nodes and number of elements (tetrahedral or triangular) were calculated.

Specimen	Oks001	Oks002	Oks003	Oks004	Oks006	Oks007	Oks008	Oks009
ACl	0.87, 0.24, 4025, 15152	0.66, 0.13, 6284, 28526	0.57,0.13, 15792, 72552	0.48, 0.09, 11726, 54489	0.42, 0.08, 15354, 76547	0.50, 0.10, 13189, 63071	0.53, 0.10, 12186, 57440	0.49, 0.09, 14018, 68169
Pcl	1.11, 0.25, 3389, 14055	0.72, 0.13, 7455, 35647	0.76,0.19, 3714, 14379	0.63, 0.15, 10769, 48710	0.62, 0.15, 10729, 48357	0.69, 0.17, 10283, 45649	0.70, 0.16, 11253, 51560	0.71, 0.19, 11793, 59303
Lcl	0.61, 0.20, 2873, 9010	0.56, 0.18, 3360, 11055	0.62,0.20, 2960, 9773	0.56, 0.12, 3408, 13306	0.40, 0.08, 8049, 32389,	0.52, 0.12, 8187, 34157	0.45, 0.09, 8630, 35687	0.43, 0.08, 9581, 41306
Mcl	0.84, 0.29, 8839, 31366	0.72, 0.30, 4661, 14465	0.55,0.16, 15693, 62712	0.42, 0.09, 19010, 83192	0.28, 0.07, 58517, 232256	0.42, 0.09, 19322, 81795	0.53, 0.10, 28897, 137254	0.36, 0.09, 18335, 74539
Mns-m	0.55, 0.13, 29608, 135176	0.30, 0.05, 94066, 477653	0.67,0.15, 11706, 51009	0.58, 0.12, 13599, 60458	0.28, 0.05, 99589, 512314	0.33, 0.06, 93452, 475512	0.42, 0.09, 63431, 310827	0.36, 0.07, 55857, 273901
Mns-l	0.57, 0.17, 20222, 90511	0.35, 0.08, 69661, 337649	0.71, 0.18, 10901, 44953	0.28, 0.06, 58394, 277929	0.34, 0.07, 72444, 356524	0.34, 0.07, 79725, 400518	0.41, 0.10, 51494, 238358	0.33, 0.07, 74632, 368347
Fmc	1.14, 0.36, 27908, 103027	1.10, 0.47, 19722, 62819	1.034,0.45, 24870, 87072	0.64, 0.24,62635,220179	0.99, 0.36, 25165, 85120	1.13, 0.36, 23148, 84098	1.19, 0.42, 21670,73673	1.12, 0.42, 25612, 86456
Ptc	0.62, 0.12, 33063, 157775	0.42, 0.12, 48109, 225560	0.62,0.12, 26121, 121105	0.47, 0.10, 39329, 179404	0.48, 0.14, 33656, 136808	0.56, 0.12, 42881, 198903	0.58, 0.14, 48539,220942	0.55, 0.13, 43103, 193241
Tbc-m	0.75, 0.16,	0.42,	0.57,0.13,	0.41, 0.12,	0.39, 0.10,	0.52, 0.15,	0.58, 0.17,	0.45, 0.12, 43536, 191271

	16193, 70116	0.12, 36242, 148206	18060, 75627	35360, 145241	48114, 214978	29169, 119930	30035, 119206	
Tbc-l	0.69, 0.15, 22878, 106371	0.47, 0.12, 35137, 155235	0.42,0.08, 40669, 200079	0.36, 0.10, 34350, 137229	0.42, 0.09, 48458, 229876	0.47, 0.12, 39497, 173275	0.57, 0.16, 39778, 168556	0.48, 0.12, 47239, 211345
Qat	0.90, 0.24, 12860, 55421	0.57, 0.12, 82238,422315	1.10,0.21, 14963, 69410	0.91, 0.18, 17462, 82857	0.55, 0.10, 72400, 363834	0.55, 0.12, 71417, 360181	0.50, 0.10, 115125, 615494	0.41, 0.06, 55870, 266284
ptl	1.06, 0.25, 11464, 48975	0.52, 0.13, 54739, 257643	1.07, 0.30 9280, 35616	0.62, 0.12, 28998, 136496	0.53, 0.14, 51502, 238304	0.58, 0.16, 62126, 320541	0.68, 0.22, 50954, 255236	0.48, 0.08, 55166, 259878
fmb	0.64, 0.13, 76178, 15232	0.95, 0.11, 24302,48600	20171, 40338,	1.02, 0.16, 20414, 40824	1.14, 0.18, 17255, 34506	1.09 0.18, 21872, 43740	1.33, 0.21, 17012, 34020	1.14, 0.18, 21629, 43254
tbb	.98, .10,21386, 42768	0.85, 0.10, 24059, 48114	0.85, 0.10, 20900, 41796	0.87, 0.15, 20171, 40338	0.76, 0.10, 24059, 48114	1.01, 0.16, 18956, 37908	1.08, 0.19, 17741, 35478	0.95, 0.15, 21629,43254
ptb	0.62, 0.07, 10802, 21600	0.61, 0.10, 9290, 18576	0.74, 0.09, 8642, 17280	0.60, 0.08, 9614, 19224	0.60, 0.10, 9722, 19440	0.68, 0.10, 9722, 19440	0.75, 0.12, 9614, 19224	0.48, 0.07, 21872, 43740
fbb	1.06, .12, 4802, 9600	0.87, 0.10, 4802, 9600	0.90, 0.12, 3794, 7584	0.37, 0.06, 20170, 40338	0.39, 0.06, 20900, 41796	0.45, 0.07, 17012, 34020	0.47, 0.08, 21872, 43740	0.44, 0.07,21143, 42282

A6. Model specific measures for convergence

Once the models were customized, some model specific adjustments had to be made for full convergence. Models were run in FeBio 2.9. If run in other versions, due to inherent software behavior, other measures might be needed to achieve convergence.

oks001 – Stiffness for springs between medial meniscus and MCL was reduced from 1000 N/mm to 300 N/mm for full convergence.

oks002, oks004, oks008 – Stiffness for springs between medial meniscus and MCL was reduced from 1000 N/mm to 3 N/mm for full convergence.

oks006 – Adjusted ties for MCL-femur. Stiffness for springs between medial meniscus and MCL was reduced from 1000 N/mm to 3 N/mm, MCL prestrain reduced to 0.025 and, LCL prestrain reduced to 0.02.

oks007 – Stiffness for springs between medial meniscus and MCL was reduced from 1000 N/mm to 3 N/mm and MCL prestrain updated to 0.025 for full convergence.

oks009 – Stiffness for springs between medial meniscus and MCL was reduced from 1000 N/mm to 3 N/mm, updated MCL- Femur ties for full convergence.

Note – check FeBio_custom.feb model file for inactive contact definitions for each model.

A7. Database folder structure

Various model related data are organized in subfolders under folder with the specimen name.

```
├── oks00x
│   ├── segmentation
│   ├── geometry
│   │   └── densities
│   ├── template model
│   │   └── FeBio
│   │   └── MED
│   ├── final model
│   │   └── processed results
├── src
├── doc
```

oks00x – Subfolders contain image segmentation labels (.nii) for all model components and registration markers (not included in the models provided) , raw and smoother geometries (used in models, .stl), additional surface meshes at various densities (.stl), volume meshes with set definitions (.med), template model (.feb), and customized final end point model (90◦ passive flexion) (.feb) with simulation result files and processed simulation kinetics –kinematics.

src – Folder contains all Python scripts used for various processes such as volume mesh generation, template model generation, model customization etc. The 'readme.txt' file and Appendix A8 describe the script usage.

doc – Folder contains all source specification documentation detailing model development and calibration. The documents with 'specifications' in the file name are the main documents describing all intended detailed processes and documents with 'deviations' in the file name contain any modifications made to the planned specifications. The 'readme.txt' file describes each of the files included in the folder. All the data were checked for completeness. Patella registration markers are missing for oks004.

Note: surface geometries for registration markers are provided for specimens oks001 and oks003 if users wish to perform registration to experimental coordinate system. If newer versions of 3D Slicer are used for surface mesh generation, there might be a coordinate system mismatch between segmentation and surface geometries.

A8. Python scripts and usage

1. StltoMed.py, MedtoFebio.py, ConnectivityXML.py

Python scripts to convert surface geometries to volume meshes and using them create template Febio models. Platform requirement and usage is as follows,

Linux

- Need Salome 7.8 to run StlToMed.py and MedToFebio.py (both written in Python 2.7)

Windows

- Need Salome 8.3 (to run MedToFebio.py, older versions do not work)
- However, if Salome 8.3 is used to correct ties and contacts in MED files then the exported version is MED 3.3.1 which will not be compatible with Salome 7.8 (required for Linux).
- In that case either use Salome 7.8 or Salome 9.2 to correct MED files and save in MED version 3.2.
- To run StlToMed.py and MedToFebio.py from Salome 8.3,

Usage -
1. Open command prompt
2. Navigate to Salome directory,
3. Type: >run_salome.bat -t --pinter path\StlToMed.py OR path\MedToFebio.py args:path\Connectivity.xml

Both

- In connectivity.xml file all .stl locations have to be pointed to the directory they are located in.

MedToFebio.py

- MED files have to be in version 3.2.
- Does not work well on Windows.

Note: **Use Febio version 2.9 as it handles contacts better**

OS preference for other software components in the pipeline:

- Segmentation/geometry generation (Slicer, Meshlab): Windows/Linux
- StltoMed.py (Salome): Linux
- Fix contacts and ties(Salome): Linux, Windows (have to export MED files in v3.2)
- MedtoFebio.py (Salome): Linux, Windows
- Pick Landmarks (Meshlab): Windows/ Linux
- Customization: Windows/Linux
- Febio: Windows/Linux (edit geometry file location in Febio_custom.feb)

2. FebCustomization_p3.py, Anatomical_Landmarks_p3.py

- Python scripts to customize template FeBio model file for material properties, loading and boundary conditions etc. (set for 90◦ passive flexion).
- When running customization for the registered models, the registered landmarks have been hard coded into a function in AnatomicalLandmarks_p3.py. In DoCalculations(), comment line 1256, and uncomment line 1259 (before running the customization script). Instead of calculating the joint axes from the manually chosen landmarks, it uses the experimentally probed landmarks, registered to the model coordinate system.
- If there are any changes in registration of the knees, the hard coded landmarks should be updated in the respective function as needed.

3. Register_probed_points.py

- This function performs the registration for open knees. To run open knees registration, go the main function at the end of the file, change the local directories for the state file and the directory containing the .stl registration markers. Run the open_knee_registration() function.
- Resulting transformation matrix, transformed anatomical landmarks will be printed to the screen. Resulting transformation matrix, transformed anatomical landmarks will be added to an xml file with registration result and saved in the current working directory.

4. tdms_processing_oks.py

- Python script used to process the tdms files from open knees data. Saves the data as a png graphs and csv files. plot_groups function can be used to save the csv, png files in intermediate steps (raw, partially processed, etc).

5. experiment_to_model.py

- This script was designed to take processed experimental data (kinematics in JCS with model offsets already removed, kinetics as forces/moments applied to the femur defined in the tibia coordinate system), and the model that results from running the customization script, and, create a model that applies to experimental conditions to the model by first converting the kinetics data into the image coordinate system, and then applying those kinetics as external forces to the femur.
- Kinematics load curves were also added, so that the kinematics can be easily applied to the cylindrical joint by setting the translation/rotation of the joint to follow the load curve. The make_model function will do this for one model. NOTE - it will change the existing febio_file if name is not given make_model(modelprops_file, febio_file, kinetics_csv, kinematics_csv, name=none) To run it for several files at the same time, read_from_xml(exp_to_mod_xml) function should be used.

6. InSituOptimization.py

- This script was designed to perform calibration of the ACL, PCL , LCL, MCL in situ strains. The input for this function is an xml file that points to the models used in the calibration, and the calibration settings. Sample xml file is provided in 'src' folder in, https://simtk.org/plugins/datashare/?group_id=485# (OKS_model) – DOI pending.
- Under the Files section: provide the path to the model properties file. The model properties file should include the added anatomical landmarks from running the customization script, as the joint axes which are added are used to process the model kinematics.
- Under the Options section: rms_error type can be defined as "loading" or "all". loading = minimize rms error in the loading direction only (ex for ACL find in situ strain to only minimize mismatch on anterior translation axis) all= minimize rms error on all 6 dof (ax for ACL in situ strain to minimize mismatch on all translation and rotation axes). opt type can be defined as "single" or "multi" . single = single variable optimization, optimize in situ strain for one ligament at a time. multi = multi variable optimization, optimize in situs train for all ligament simultaneously (this will take much longer)
- Under the Ligaments section: for each of the ligaments, provide the febio file (created using experiment_to_model.py). For example, for the ACL, this would be a model generated to mimic the 0 degree flexion anterior loading test. (doesn't need to be 0 degrees, we just decided 0 because the models run the fastest) provide the experiment kinematics file - needs to match with whatever file was used for the febio file. For example, if ACL 0 degree anterior

laxity, then this should be the kinematics file for that trial in situ strain is the initial guess for in situ strain. This shouldn't matter in theory - eventually it should find the solution.

- However, if the optimization has already run and a small change is made and need to run it again to be sure it doesn't affect results, etc. a known solution can be put as the initial guess to save a lot of computation time.
- The order in which these ligaments appear in the xml file is the order in which they will be optimized. Running this script will create summary files named Optimization<num>.txt, where num is the counter for which round of optimization it is on.
- The script will continue to loop on all the ligaments until convergence is achieved. Essentially create febio models for each laxity test (using experiment_to_model.py) and put them each in their own folder. Then create the InSituOpt.xml file pointing to each of these files, and other relevant files. Then run from the terminal as:
  python InSituOptimization.py /path/to/InSituOpt.xml febioCommand

  febioCommand is the path to the febio execulatble, on linux it can be found in
  '/FEBio2.8.2/bin/febio2.lnx64'

  If always running from the same computer, the febio command can be hard coded into the run_from_xml(). Leave out the febioCommand when running from the terminal.

7. LogPostProcessing.py

- Python script creates a folder of images and csv files with model results. Can be run using Exp_to_Mod.xml with the run_all_in_file() function.

8. model_prediction_errors.py

- To run after LogPostProcessing.py. Compares the kinematics between the model results and the experiment kinematics. Creates images, and files storing the rms error between them. Can be run using Exp_to_Mod.xml with the from_xml() function. see main function at end of script for examples of use.

9. transfer_med_groups.py

- Transfers nodes and element sets from one mesh to another for a given component by treating the groups as a binary field and applying that binary field to the new mesh to find the correspondence of nodes and elements in the new mesh. This correspondence is used to select nodes and elements to generate groups with the new node and element sets.

10. StiffnessFromLog.py

- Python script extracts rigid body reaction force – displacement data from simulation output (.log).

11. ModelReduction_rigids3.py

- Removes all contacts and ties for the specified component that needs to be removed and converts it into a rigid body.

12. edge_lengths.py

- Python script to calculate average edge lengths, standard deviations, minimum and maximum edge lengths for a given volume mesh (.med).

Note: Full path of Geometry.feb file has to be provided in the FeBio_custom.feb file if using Febio 2.9 in Windows. If using Linux, there is no need to supply the whole path as long as the files are in the same folder.

References

1. 3D Slicer image computing platform. 3D Slicer. https://slicer.org/

2. Aframian A, Smith TO, Tennent TD, Cobb JP, Hing CB. Origin and insertion of the medial patellofemoral ligament: a systematic review of anatomy. *Knee Surg Sports Traumatol Arthrosc*. 2017;25(12):3755-3772. doi:10.1007/s00167-016-4272-1

3. *BioRobotics, SimVitro - SimVITRO Data File Contents for Open Knee, User Manual, 2013CB-031-002.B.* https://simtk.org/plugins/moinmoin/openknee/Infrastructure/ExperimentationMechanics?action=AttachFile&do=get&target=2013CB-031-002.B+simVITRO+Data+File+Contents_Open+Knee.pdf

4. *BioRobotics, SimVitro - State Configuration File Explanations, User Manual, 2013CB-031-001.A.* https://simtk.org/plugins/moinmoin/openknee/Infrastructure/ExperimentationMechanics?action=AttachFile&do=get&target=2013CB-031 001.A_State+Configuration+File+Explanations.pdf

5. Bull AMJ, Katchburian MV, Shih YF, Amis AA. Standardisation of the description of patellofemoral motion and comparison between different techniques. *Knee Surg Sports Traumatol Arthrosc*. 2002;10(3):184-193. doi:10.1007/s00167-001-0276-5

6. Chokhandre S, Neumann EE, Nagle TF, et al. Specimen specific imaging and joint mechanical testing data for next generation virtual knees. *Data Brief*. 2021;35:106824. doi:10.1016/j.dib.2021.106824

7. Cignoni P, Callieri M, Corsini M, Dellepiane M, Ganovelli F, Ranzuglia G. MeshLab: an Open-Source Mesh Processing Tool. *Eurographics Italian Chapter Conference*. Published online 2008:8 pages. doi:10.2312/LOCALCHAPTEREVENTS/ITALCHAP/ITALIANCHAPCONF2008/129-136

8. dfwg. NIfTI: — Neuroimaging Informatics Technology Initiative. https://nifti.nimh.nih.gov/

9. Dhaher YY, Kwon TH, Barry M. The effect of connective tissue material uncertainties on knee joint mechanics under isolated loading conditions. *J Biomech*. 2010;43(16):3118-3125. doi:10.1016/j.jbiomech.2010.08.005

10. Donahue TLH, Hull ML, Rashid MM, Jacobs CR. A finite element model of the human knee joint for the study of tibio-femoral contact. *J Biomech Eng*. 2002;124(3):273-280. doi:10.1115/1.1470171

11. FEBio Software Suite. https://febio.org/

12. FEBio Theory Manual. FEBio Documentation. https://help.febio.org/FEBioTheory/FEBio_tm_3-4.html

13. FEBio User Manual Version 2.9. https://help.febio.org/FEBio/FEBio_um_2_9/index.html

14. Gardiner JC, Weiss JA. Subject-specific finite element analysis of the human medial collateral ligament during valgus knee loading. *J Orthop Res*. 2003;21(6):1098-1106. doi:10.1016/S0736-0266(03)00113-X

15. Grood ES, Suntay WJ. A joint coordinate system for the clinical description of three-dimensional motions: application to the knee. *J Biomech Eng*. 1983;105(2):136-144. doi:10.1115/1.3138397

16. *Knee Joint Coordinate System v2.1*. https://simtk.org/plugins/moinmoin/openknee/Infrastructure/ExperimentationMechanics?action=AttachFile&do=get&target=Knee+Coordinate+Systems.pdf

17. Lechner K, Hull ML, Howell SM. Is the circumferential tensile modulus within a human medial meniscus affected by the test sample location and cross-sectional area? *J Orthop Res*. 2000;18(6):945-951. doi:10.1002/jor.1100180614

18. Maas SA, Erdemir A, Halloran JP, Weiss JA. A general framework for application of prestrain to computational models of biological materials. *Journal of the Mechanical Behavior of Biomedical Materials*. 2016;61:499-510. doi:10.1016/j.jmbbm.2016.04.012

19. Netgen/NGSolve. https://ngsolve.org/

20. Office Open XML - What is OOXML? http://officeopenxml.com/

21. Peña E, Calvo B, Martínez MA, Doblaré M. A three-dimensional finite element analysis of the combined behavior of ligaments and menisci in the healthy human knee joint. *J Biomech*. 2006;39(9):1686-1701. doi:10.1016/j.jbiomech.2005.04.030

22. PostView – FEBio Software Suite. https://febio.org/postview/

23. Roelofs G. PNG: The Definitive Guide.

24. SALOME MED Users' Guide: MEDMEM library. https://docs.salome-platform.org/5/med/user/medmem.html

25. SALOME NETGENPLUGIN User's Guide: NETGEN 2D and 3D hypotheses. https://docs.salome-platform.org/latest/gui/NETGENPLUGIN/netgen_2d_3d_hypo_page.html

26. Salome Platform - The open-source platform for numerical simulation. https://www.salome-platform.org/

27. Shafranovich Y. Common Format and MIME Type for Comma-Separated Values (CSV) Files. Published online 2005. https://www.rfc-editor.org/info/rfc4180

28. Shriram D, Praveen Kumar G, Cui F, Lee YHD, Subburaj K. Evaluating the effects of material properties of artificial meniscal implant in the human knee joint using finite element analysis. *Sci Rep*. 2017;7(1):6011. doi:10.1038/s41598-017-06271-3

29. STL (STereoLithography) File Format Family. Published September 12, 2019. https://www.loc.gov/preservation/digital/formats/fdd/fdd000504.shtml

30. Thambyah A, Nather A, Goh J. Mechanical properties of articular cartilage covered by the meniscus. *Osteoarthr Cartil*. 2006;14(6):580-588. doi:10.1016/j.joca.2006.01.015

31. The NI TDMS File Format - National Instruments. http://www.ni.com/product-documentation/3727/en/

32. Welcome to Python.org. Python.org. https://www.python.org/
